# Hydroxyl radicals generated by hydrogen peroxide photolysis recondition biofilm-contaminated titanium surfaces for subsequent osteoblastic cell proliferation

**DOI:** 10.1038/s41598-019-41126-z

**Published:** 2019-03-18

**Authors:** Keisuke Nakamura, Midori Shirato, Taichi Tenkumo, Taro Kanno, Anna Westerlund, Ulf Örtengren, Keiichi Sasaki, Yoshimi Niwano

**Affiliations:** 1 Department of Advanced Free Radical Science, Tohoku University Graduate School of Dentistry, 4-1 Seiryo, Aoba-ku, Sendai 980-8575 Japan; 2 Department of Orthodontics, Institute of Odontology, Sahlgrenska Academy, University of Gothenburg, Gothenburg, 40530 Sweden; 3 Department of Clinical Dentistry/Faculty of Health Sciences, The Arctic University of Norway, Tromsø, 9037 Norway; 4 Department of Cariology, Institute of Odontology, Sahlgrenska Academy, University of Gothenburg, Gothenburg, 40530 Sweden; 5 Division of Advanced Prosthetic Dentistry, Tohoku University Graduate school of Dentistry, 4-1 Seiryo, Aoba-ku, Sendai 980-8575 Japan; 6 Faculty of Nursing, Shumei University, 1-1 Daigaku-cho, Yachiyo, Chiba 276-0003 Japan

## Abstract

Titanium dental implants have been successfully used for decades; however, some implants are affected by peri-implantitis due to bacterial infection, resulting in loss of supporting bone. This study aimed to evaluate the effect of an antimicrobial chemotherapy employing H_2_O_2_ photolysis—developed to treat peri-implantitis—on biofilm-contaminated titanium surfaces in association with osteoblastic cell proliferation on the treated surface. Titanium discs were sandblasted and acid-etched, followed by contamination with a three-species biofilm composed of *Porphyromonas gingivalis*, *Fusobacterium nucleatum*, and *Streptococcus mitis*. This biofilm model was used as a simplified model of clinical peri-implantitis biofilm. The discs were subjected to ultrasound scaling, followed by H_2_O_2_ photolysis, wherein 365-nm LED irradiation of the disc immersed in 3% H_2_O_2_ was performed for 5 min. We analysed proliferation of mouse osteoblastic cells (MC3T3-E1) cultured on the treated discs. Compared with intact discs, biofilm contamination lowered cell proliferation on the specimen surface, whereas H_2_O_2_ photolysis recovered cell proliferation. Thus, H_2_O_2_ photolysis can recover the degraded biocompatibility of biofilm-contaminated titanium surfaces and can potentially be utilised for peri-implantitis treatment. However, to verify the findings of this study in relation to clinical settings, assessment using a more clinically relevant multi-species biofilm model is necessary.

## Introduction

Titanium dental implants establish osseointegration^1^ and have been widely used to treat partial and complete edentulism with relatively high survival rates^2–4^. However, some implants become affected by peri-implantitis over time^5^, which is an inflammatory disease caused by pathogenic microorganisms that degrade osseointegration (i.e. loss of supporting bone around implants)^6,7^. As microbial biofilm growth on the implant surface is the major etiologic agent of peri-implantitis^8,9^, effective elimination and/or inactivation of the biofilm is crucial. However, modern dental implants have a screw-shaped design for post-installation stability and thus, mechanical instrumentation is difficult to implement and insufficient to reduce bacterial load—especially at the valley of the threads—which leaves biofilms on the surface^10–12^. In addition, the rough surfaces of the implant facilitate osseointegration but may deter biofilm elimination^12^. Therefore, it is recommended that mechanical instrumentation be accompanied with additional chemical decontamination^11–13^.

For the decontamination of titanium implant surfaces, several antiseptic medications, including citric acid, povidone-iodine (PI), chlorhexidine gluconate (CHX), and hydrogen peroxide (H_2_O_2_), have been used clinically^11,12,14^. Because treatment is aimed to arrest progression of peri-implantitis and ideally achieve re-osseointegration, requirements for antimicrobial chemotherapy are to effectively eliminate or kill biofilm bacteria and to recondition the titanium surface for subsequent osteoblast proliferation. However, conventional antiseptics cannot completely remove biofilm constituents containing carbon and nitrogen elements even if they can reduce bacterial load^15^. Contaminants such as hydrocarbons left on titanium surfaces may reduce osteoblast attachment to the surface because proteins associated with cell adhesion cannot attach to such sites^16,17^. Moreover, some antiseptics alter the physical and/or chemical properties of titanium surfaces, resulting in less favourable conditions for osteoblast proliferation and re-osseointegration^15,18,19^. A systematic review suggested that data on the efficacy of chemotherapeutic agents on biofilm-contaminated titanium are scarce and that a definitive conclusion is unattainable^20^. Thus, to date, no single antimicrobial treatment has been proven superior with respect to the requirements.

A novel antimicrobial technique where hydroxyl radicals generated by H_2_O_2_ photolysis serve as the active ingredient has been developed^21^. The technique relies on hydroxyl radicals being powerful oxidising agents that cause lethal oxidative damage to microorganisms. However, hydroxyl radicals cannot be formulated as a ready-made disinfectant owing to their very short half-life in liquid (approximately 10^−9^ s)^22^ and are instead generated by irradiating 3% H_2_O_2_ at the lesion site with 365–405 nm light (i.e. photolysis)^21,23^. Because the light and H_2_O_2_ can penetrate the microbial biofilm, this antimicrobial technique is also effective against biofilm bacteria^24^. Compared with conventional antiseptics used in oral cavities such as 0.2% CHX, 0.5% PI, and 3% H_2_O_2_, antimicrobial action by H_2_O_2_ photolysis exerts much higher bactericidal activity against biofilm bacteria^25^. Furthermore, a randomised controlled clinical trial recently reported that this technique can be used as an adjunctive antimicrobial chemotherapy for periodontitis treatment as it significantly decreases the amount of pathogenic bacteria and improves periodontal condition^26^. Thus, this antimicrobial technique is also expected to be applicable for peri-implantitis treatment. However, the effect of this technique on biofilm-contaminated titanium surfaces and subsequent osteoblastic cell proliferation remains unclear. Therefore, the present study aimed to evaluate the effect of H_2_O_2_ photolysis on the chemical condition of biofilm-contaminated titanium surfaces and to assess osteoblastic cell proliferation on the treated surfaces. Furthermore, we evaluated the potential beneficial and adverse effects of this treatment by examining physical, chemical, and biological properties of newly prepared (intact) and aged titanium surfaces subjected to H_2_O_2_ photolysis.

## Results

### Effect of H_2_O_2_ photolysis on titanium surfaces without biofilm contamination

**Figure 1:**
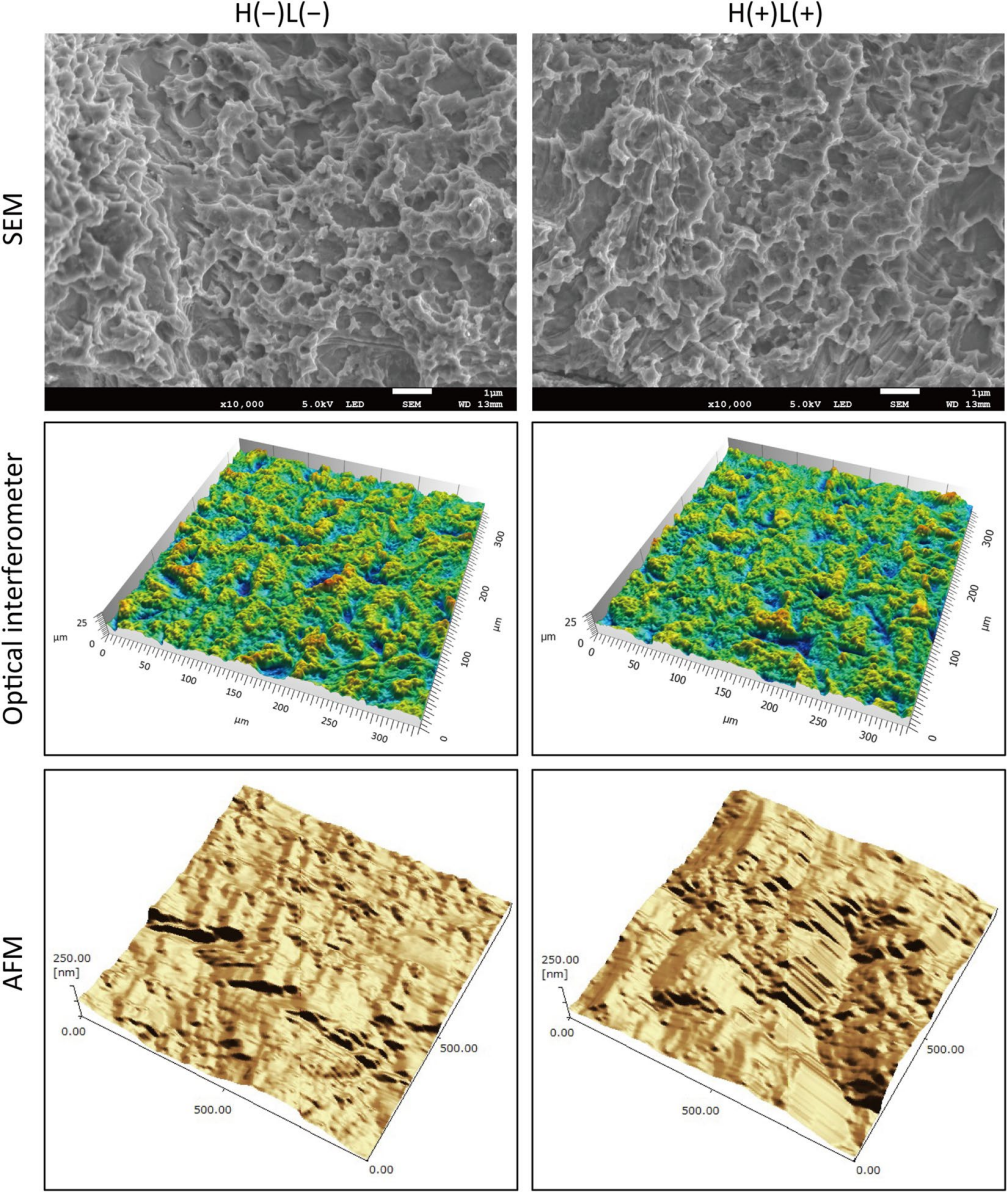
Representative microscopic images of titanium surfaces treated with or without H_2_O_2_ photolysis. Commercially pure titanium discs (5 mm in diameter and 2-mm thick) were sandblasted and acid-etched to obtain a rough surface similar to that commonly used in commercial dental implants. The titanium discs were treated with either pure water alone [H(−)L(−)] or photolysis of 3% H_2_O_2_ by 365-nm LED irradiation [H(+)L(+)]. Microscopic examination using a scanning electron microscope (SEM), an optical interferometer, and an atomic force microscope (AFM) showed similar micro- and nano-scale structures for both groups. SEM scale bar = 1 µm. The field views for optical interferometry and AFM analysis were 336 × 336 µm and 1 × 1 µm, respectively.

Microscopic images of newly prepared titanium discs (New-Ti) treated for 5 min with LED irradiation of 3% H_2_O_2_ at a wavelength of 365 nm (H_2_O_2_ photolysis) denoted as H(+)L(+) and those treated with pure water without LED irradiation denoted as H(−)L(−) showed similar micro- and nano-structural attributes at the titanium surface (Fig. 1). Surface analysis using an optical interferometer and atomic force microscope (AFM) revealed that H(+)L(+) treatment did not significantly affect the micro- or nano-scale surface roughness of New-Ti (*p* > 0.05; Table 1).

**Table 1:** Surface roughness of titanium discs treated with or without H_2_O_2_ photolysis (n = 6).

			Sa (µm)	Sq (µm)	Sp (µm)	Sv (µm)	Sz (µm)	Sdr (%)	Sds (1/µm^2^)
Micro- scale	H(−)L(−)	Ave	1.97	2.48	10.01	12.78	22.79	40.07	0.07
		SD	0.08	0.09	1.19	1.41	2.00	1.20	<0.01
	H(+)L(+)	Ave	2.00	2.53	10.00	11.46	21.45	40.47	0.07
		SD	0.05	0.07	1.32	1.34	2.63	1.65	<0.01
			p > 0.05	p > 0.05	p > 0.05	p > 0.05	p > 0.05	p > 0.05	p > 0.05
			**Sa (nm)**	**Sq (nm)**	**Sp (nm)**	**Sv (nm)**	**Sz (nm)**	**—**	**—**
Nano- scale	H(−)L(−)	Ave	30.63	40.22	114.71	138.67	253.38	—	—
		SD	11.12	14.15	47.64	41.70	82.46	—	—
	H(+)L(+)	Ave	31.87	40.94	146.76	137.79	284.55	—	—
		SD	11.05	13.84	66.62	26.76	85.49	—	—
			p > 0.05	p > 0.05	p > 0.05	p > 0.05	p > 0.05	—	—

Ave, average; SD, standard deviation; Sa, arithmetic average; Sq, root mean squared; Sp, maximum peak height; Sv, maximum valley depth; Sz, peak to valley; Sdr, increment of the interfacial surface area to a flat plane baseline; Sds, density of summits.

X-ray diffraction (XRD) analysis showed that both H(−)L(−) and H(+)L(+)-treated New-Ti comprised titanium (Ti), titanium hydride (TiH_2_), and titanium dioxide [TiO_2_ (anatase); Fig. S1]. Debye diffraction cones observed in the 2-dimensional (2D) diffraction images confirmed that a small TiO_2_ peak at 25.5° resulted from diffraction. There was no significant difference in peak intensities between H(−)L(−) and H(+)L(+)-treated New-Ti (*p* > 0.05).

**Figure 2:**
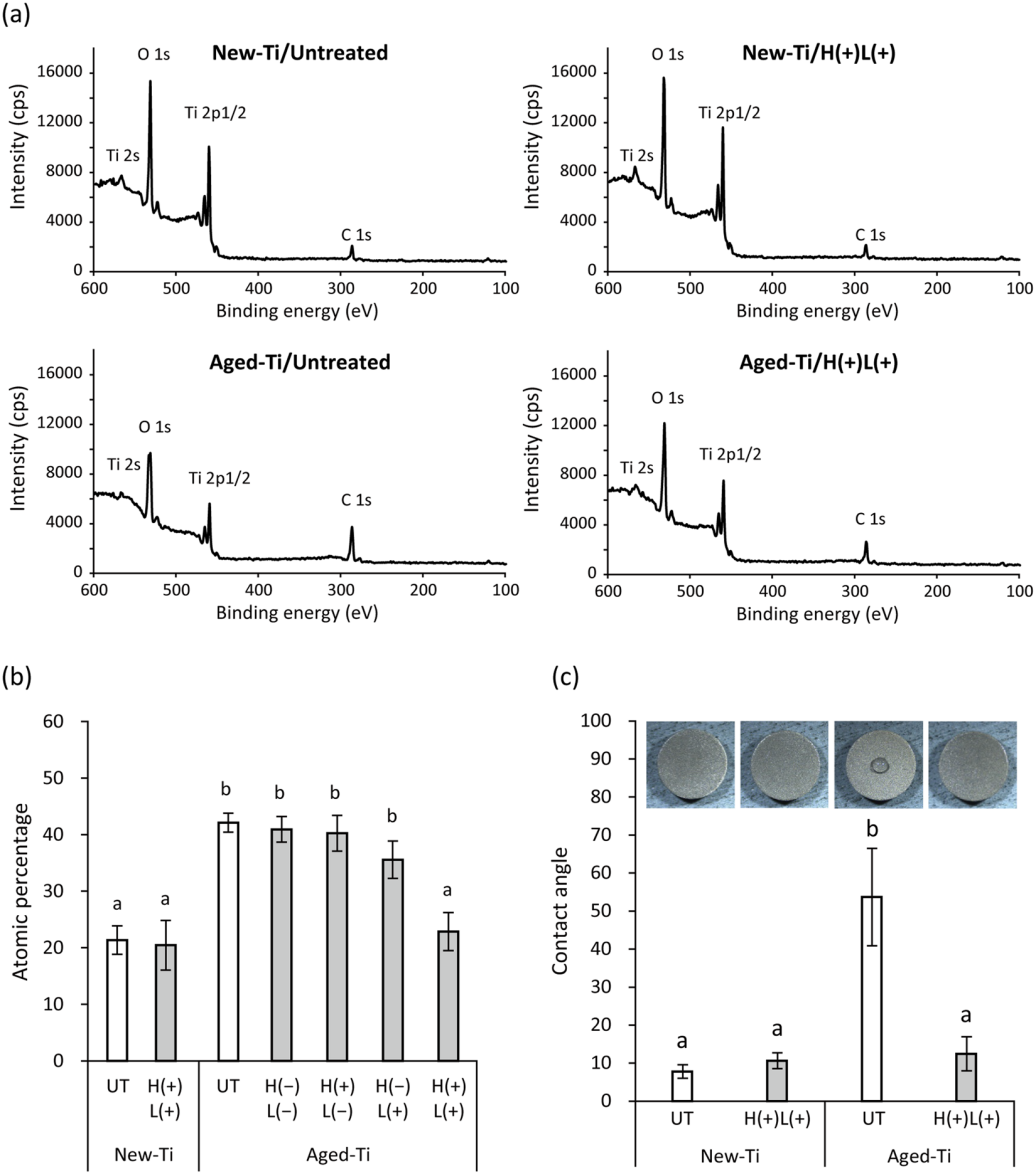
Surface conditions of newly prepared (New-Ti) and aged titanium (Aged-Ti) discs treated with or without H_2_O_2_ photolysis. (**a**) Representative X-ray photoelectron spectroscopy spectra. (**b**) Atomic percentage of carbon on titanium specimen surfaces. (**c**) Contact angle of 0.4 µL pure water droplet on titanium surfaces and photographic images of the spread of the droplet. New-Ti and Aged-Ti were immersed in 3% H_2_O_2_ and irradiated with 365 nm LED, either alone or in combination denoted as H(−)L(−), H(+)L(−), H(−)L(+), or H(+)L(+), for 5 min. Aging increased the amount of carbon on titanium discs, converting the hydrophilic intact titanium surface to hydrophobic. H(+)L(+) treatment significantly reduced the amount of carbon on Aged-Ti and recovered surface hydrophilicity. Values and error bars in (**b**,**c**) indicate the mean and standard deviation, respectively [n = 4 for (**b**) and n = 6 for (**c**)]. Different letters above the columns in (**b**,**c**) refer to significant differences (*p* < 0.01) between different groups. UT, untreated; H(−)L(−), treatment with pure water in a light-shielding box; H(+)L(−), treatment with 3% H_2_O_2_ in a light-shielding box; H(−)L(+), 365-nm LED irradiation of sample in pure water; H(+)L(+), 365-nm LED irradiation of sample in 3% H_2_O_2_.

X-ray photoelectron spectroscopy (XPS) demonstrated the presence of titanium, oxygen, and carbon on the surface of New-Ti (Fig. 2a). When titanium specimens were stored under ambient conditions for 4 weeks after specimen preparation (Aged-Ti), the percentage of carbon significantly increased from 21% (New-Ti) to 42% (Aged-Ti; *p* < 0.01; Fig. 2b). H(+)L(+) treatment significantly decreased the percentage of carbon on Aged-Ti to 23% (*p* < 0.01) but did not affect the carbon percentage of New-Ti (*p* > 0.05). Treatment with LED irradiation alone [H(−)L(+)] slightly decreased the percentage of carbon on Aged-Ti to 36%, but it was not significant (*p* = 0.08). Neither H(−)L(−) nor H(+)L(−) (immersion in 3% H_2_O_2_; no LED irradiation) treatment significantly affected the percentage of carbon on Aged-Ti (*p* > 0.05).

On the surface of New-Ti, a droplet of ultrapure water (Milli-Q® water) spread quickly indicating that the surface was hydrophilic. The contact angle of New-Ti was approximately 10° regardless of H(+)L(+) treatment (Fig. 2c). Upon aging, the surface became hydrophobic and exhibited a contact angle of >50°. H(+)L(+) treatment significantly decreased the 54° contact angle of Aged-Ti to 12° (*p* < 0.01), thereby recovering surface hydrophilicity.

### Effect of ultrasound scaling followed by H_2_O_2_ photolysis on *Aggregatibacter actinomycetemcomitans* biofilm-contaminated titanium surfaces

The viable count of *A*. *actinomycetemcomitans* biofilm formed on titanium discs (*Aa* biofilm-Ti) was 1.3 × 10^5^ colony-forming units (CFU)/specimen. Ultrasound scaling using a plastic scaler tip made of polyether ether ketone (PEEK) [US(+)H(−)L(−)] significantly decreased the number of viable bacteria by 3-log (1.5 × 10^2^ CFU/specimen; *p* < 0.01). The following 5-min treatments with any of the antimicrobial techniques tested [i.e. H(+)L(−), H(−)L(+), H(+)L(+), 0.2% CHX, or 0.5% PI] resulted in no detection of viable bacteria (detection limit = 2 CFU/specimen; Table S1).

**Figure 3:**
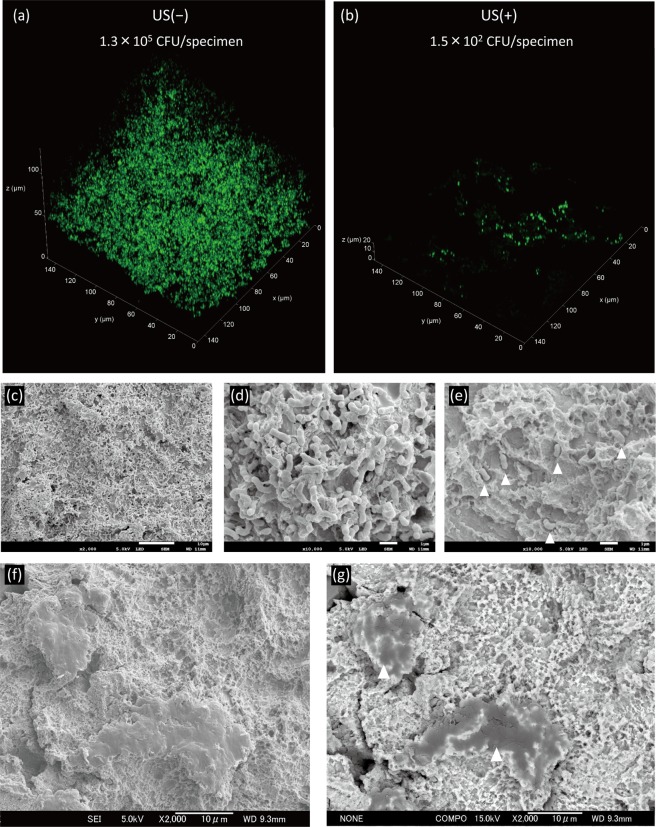
Representative confocal laser scanning microscopy and scanning electron microscopy images of *A*. *actinomycetemcomitans* biofilms formed on titanium specimens treated with or without ultrasound scaling (US). (**a**) *A*. *actinomycetemcomitans* biofilm formed on titanium specimens, and (**b**) US treatment of the biofilm at a field view of 148 × 148 µm. (**c**) Biofilm at low magnification. Scale bar = 10 µm. (**d**) Biofilm at high magnification. Scale bar = 1 µm. (**e**) Remaining bacteria after US. Scale bar = 1 µm. White arrowheads indicate bacterial cells. (f) Secondary electron image of a titanium surface after US. Scale bar = 10 µm. (**g**) Backscattered electron image of (f). Scale bar = 10 µm. White arrowheads indicate remnants of the plastic scaler tip.

Confocal laser scanning microscopy (CLSM) of *Aa* biofilms with and without ultrasound scaling showed the 3D structure of each biofilm (Fig. 3a,b). Ultrasound scaling eliminated large portions of the biofilm, although some bacteria persisted on the surface as observed by CLSM (Fig. 3b). Scanning electron microscopy (SEM) revealed that the surface of *Aa* biofilm-Ti was entirely covered with bacterial cells and extracellular matrix (Fig. 3c,d). Furthermore, SEM showed that several bacterial cells persisted in micro-pits of the titanium rough surface after ultrasound scaling (Fig. 3e). On titanium surfaces subjected to ultrasound scaling using PEEK tips, protrusions of micro-roughened surfaces that appeared collapsed were observed in secondary electron images (Fig. 3f). The backscattered electron image showed a clear contrast between the protrusions and intact titanium surfaces (Fig. 3g), indicating that the protrusions contain other material than titanium.

**Figure 4:**
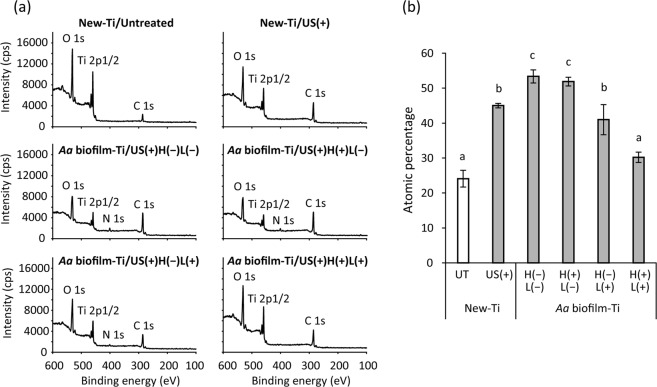
Chemical composition of *A*. *actinomycetemcomitans* biofilm-contaminated titanium (*Aa* biofilm-Ti) surfaces treated with H_2_O_2_ photolysis. (**a**) Representative X-ray photoelectron spectroscopy spectra and (**b**) atomic percentage of carbon on titanium specimen surfaces. *Aa* biofilm-Ti was subjected to ultrasound scaling (US) followed by immersion in 3% H_2_O_2_ and irradiation with 365 nm LED, either alone or in combination denoted as H(−)L(−), H(+)L(−), H(−)L(+), or H(+)L(+), for 5 min. *Aa* biofilm contamination increased the amount of carbon on titanium discs. Photolysis of 3% H_2_O_2_ by 365-nm LED irradiation, denoted as H(+)L(+), significantly reduced the amount of carbon on *Aa* biofilm-Ti. Values and error bars in (**b**) indicate the mean and standard deviation, respectively (n = 3). Different letters above the columns in (**b**) refer to significant differences (p < 0.01) between different groups. UT, untreated; H(−)L(−), treatment with pure water in a light-shielding box; H(+)L(−), treatment with 3% H_2_O_2_ in a light-shielding box; H(−)L(+), 365-nm LED irradiation of sample in pure water; H(+)L(+), 365-nm LED irradiation of sample in 3% H_2_O_2_.

XPS analysis demonstrated that New-Ti subjected to ultrasound scaling with PEEK tips significantly increased carbon percentage from 24% to 45% (*p* < 0.01; Fig. 4a,b). On the surface of ultrasound scaling and H(−)L(−)-treated *Aa* biofilm-Ti, the percentage of carbon (53%) was significantly higher than that of New-Ti treated with ultrasound scaling (*p* < 0.01). Meanwhile, both H(+)L(+) and H(−)L(+) treatments significantly reduced the percentage of carbon on *Aa* biofilm-Ti (*p* < 0.01) and the former showed a significantly lower percentage of carbon (30%) than the latter (41%; *p* < 0.01). Moreover, a small peak for nitrogen was observed on H(−)L(−)-treated *Aa* biofilm-Ti (Fig. 4a). This nitrogen peak was still detected on surfaces treated with H(+)L(−) and H(−)L(+), whereas the peak was not detected after H(+)L(+) treatment.

### Osteoblast proliferation on aged titanium surfaces

**Figure 5:**
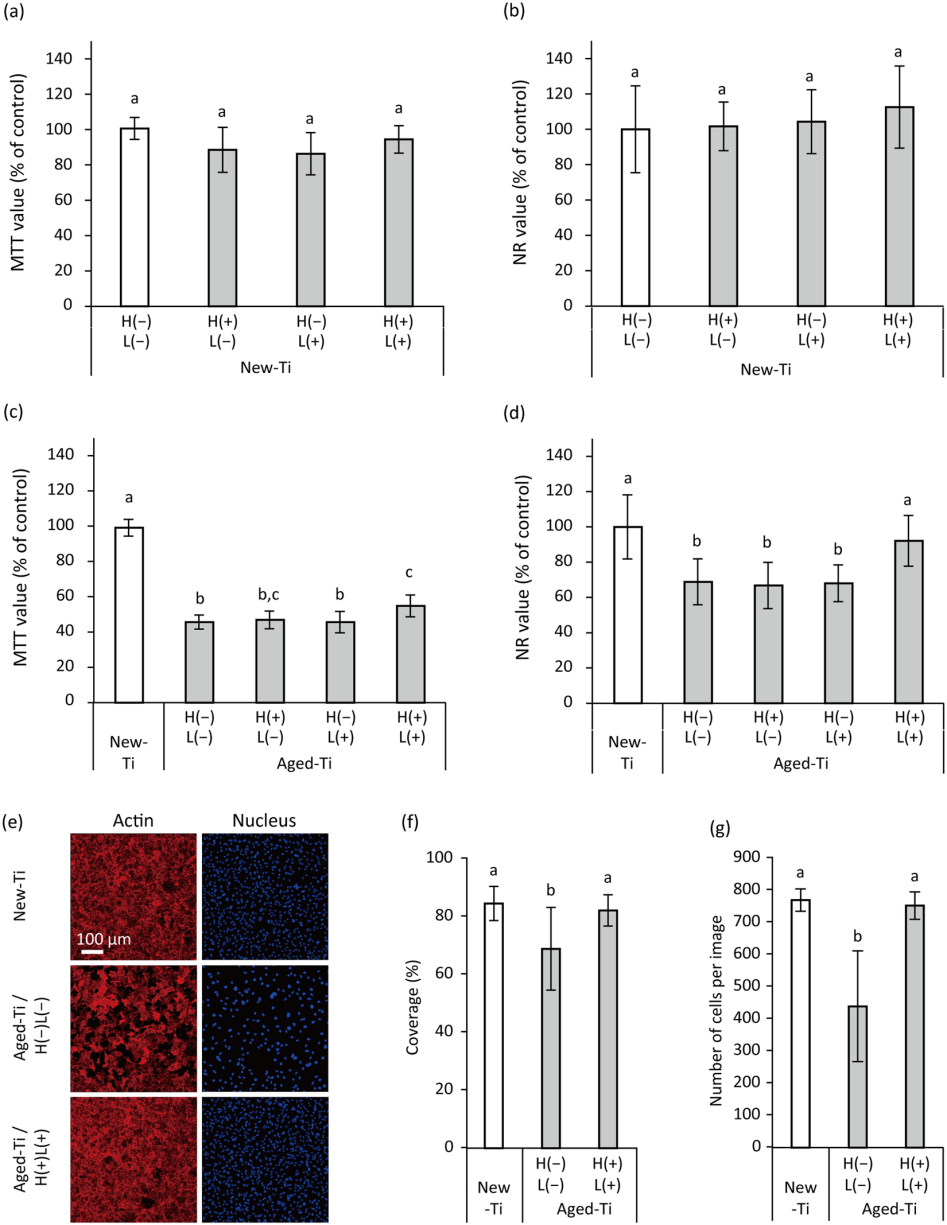
Proliferation of MC3T3-E1 osteoblastic cells cultured on H_2_O_2_ photolysis-treated newly prepared (New-Ti) and aged titanium (Aged-Ti), as assessed by methyl thiazolyl tetrazolium (MTT) assays, neutral red (NR) assays, and confocal laser scanning microscopy (CLSM). Cells were cultured on the specimens for 3 d before assays were performed. (**a**,**b**) Relative optical density values obtained in MTT assay (MTT value) and NR assay (NR values) for cells grown on New-Ti and (**c**,**d**) Aged-Ti. The titanium discs were immersed in 3% H_2_O_2_ and irradiated with 365 nm LED, either alone or in combination denoted as H(−)L(−), H(+)L(−), H(−)L(+), or H(+)L(+), for 5 min. (**e**) Representative CLSM images of MC3T3-E1 osteoblastic cells cultured on New- Ti and Aged-Ti treated with H(−)L(−) and H(+)L(+). Cell nuclei and actin filaments were stained with DAPI and rhodamine phalloidin, respectively. (**f**) Quantitative results of cellular coverage and (**g**) number of cells. Parameter values for cell proliferation on Aged-Ti were significantly lower than those for cells grown on New-Ti. However, H(+)L(+) treatment recovered the reduction in cell proliferation induced by aging. Values and error bars in the graphs indicate the mean and standard deviation, respectively [n = 6 for (**a**–**d**) and n = 9 for (**f**,**g**)]. Different letters above the columns in the graphs refer to significant differences (*p* < 0.05) between different groups. H(−)L(−), treatment with pure water in a light-shielding box; H(+)L(−), treatment with 3% H_2_O_2_ in a light-shielding box; H(−)L(+), 365-nm LED irradiation of sample in pure water; H(+)L(+), 365-nm LED irradiation of sample in 3% H_2_O_2_.

Methyl thiazolyl tetrazolium (MTT) and neutral red (NR) assays demonstrated that proliferation of the mouse osteoblastic cell line MC3T3-E1 cultured for 3 d on H(+)L(+)-treated New-Ti was not significantly different from that of cells cultured on H(−)L(−), H(+)L(−), and H(−)L(+)-treated New-Ti (*p* > 0.05; Fig. 5a,b). Aging of the titanium surface significantly reduced cell proliferation (*p* < 0.01; Fig. 5c,d). Of the treatments tested, only H(+)L(+) significantly increased the proliferation of cells cultured on Aged-Ti as evidenced by both MTT and NR assays (*p* < 0.05); however, relative optical density (OD) values obtained via MTT assays (hereafter referred to as the MTT value) of H(+)L(+)-treated Aged-Ti were still significantly lower than those of cells cultured on New-Ti (*p* < 0.01; Fig. 5c), whereas relative OD values obtained via NR assays (hereafter referred to as the NR value) of H(+)L(+)-treated Aged-Ti were comparable to those of cells cultured on New-Ti (*p* > 0.05; Fig. 5d). CLSM analysis revealed that cells were densely proliferated on New-Ti after a 3-d culture irrespective of H(−)L(−) or H(+)L(+) treatment (Fig. S2) and there were no significant differences in cellular coverage and cell number between the two groups (*p* > 0.05). Compared with New-Ti, the cell density of H(−)L(−)-treated Aged-Ti was lower than that of New-Ti (Fig. 5e); however, cell proliferation on H(+)L(+)-treated Aged-Ti was comparable to that of New-Ti (Fig. 5e). Quantitative analysis confirmed that cellular coverage and cell number of New-Ti and H(+)L(+)-treated Aged-Ti were significantly higher than those of H(−)L(−)-treated Aged-Ti (*p* < 0.05), while there was no significant difference between New-Ti and H(+)L(+)-treated Aged-Ti (*p* > 0.05; Fig. 5f,g). Regarding cell morphometric analyses, there were no significant differences in the area (cell size), Feret’s diameter, and perimeter of cells grown on New-Ti, H(−)L(−)-treated Aged-Ti, and H(+)L(+)-treated Aged-Ti (*p* > 0.05; Fig. S3).

### Osteoblast proliferation on *A*. *actinomycetemcomitans* biofilm-contaminated titanium surfaces

**Figure 6:**
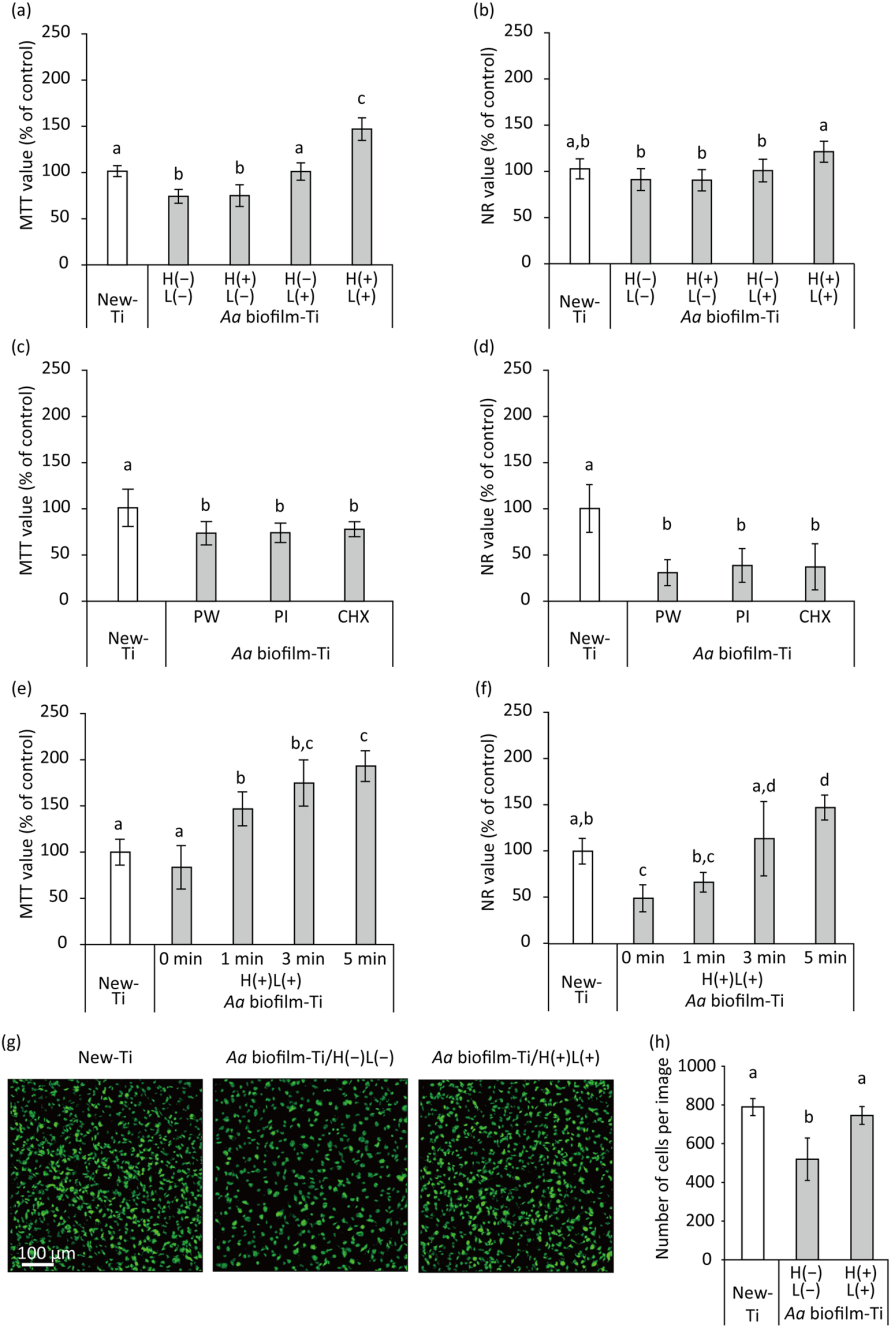
Proliferation of MC3T3-E1 osteoblastic cells cultured on *A*. *actinomycetemcomitans* biofilm-contaminated titanium (*Aa* biofilm-Ti) treated with H_2_O_2_ photolysis, as assessed by methyl thiazolyl tetrazolium (MTT) assays, neutral red (NR) assays, and confocal scanning laser microscopy (CLSM). *Aa* biofilm-Ti was subjected to ultrasound scaling followed by each treatment and then cells were cultured on the treated specimens for 3 d before the assays were performed. (**a**,**b**) Relative optical density values obtained via MTT (MTT value) and NR (NR values) assays for cells grown on *Aa* biofilm-Ti that were immersed in 3% H_2_O_2_ and irradiated with 365 nm LED, either alone or in combination denoted as H(−)L(−), H(+)L(−), H(−)L(+), or H(+)L(+), for 5 min. (**c**,**d**) Relative MTT and NR values for cells grown on *Aa* biofilm-Ti treated with pure water (PW), 0.5% (w/v) povidone-iodine (PI), and 0.2% (w/v) chlorhexidine gluconate (CHX). (**e**,**f**) Relative MTT and NR values for cells grown on *Aa* biofilm-Ti treated with H(+)L(+) for 1, 3, and 5 min. (**g**) Representative CLSM images of MC3T3-E1 osteoblastic cells cultured on New-Ti and *Aa* biofilm-Ti treated with H(−)L(−) and H(+)L(+) for 5 min. Cell nuclei were stained with SYTO9. (**h**) Cell quantification. Parameter values for cell proliferation on *Aa* biofilm-Ti were significantly lower than those for cells grown on New-Ti. However, H(+)L(+) treatment recovered the reduction in cell proliferation induced by *Aa* biofilm contamination. Values and error bars in the graphs indicate the mean and standard deviation, respectively [n = 6 for (**a**–**f**) and n = 9 for (**h**)]. Different letters above the columns in the graphs refer to significant differences (*p* < 0.05) between different groups. H(−)L(−), treatment with pure water in a light-shielding box; H(+)L(−), treatment with 3% H_2_O_2_ in a light-shielding box; H(−)L(+), 365-nm LED irradiation of sample in pure water; H(+)L(+), 365-nm LED irradiation of sample in 3% H_2_O_2_.

MC3T3-E1 cells cultured for 3 d on H(−)L(−)-treated *Aa* biofilm-Ti showed significantly lower MTT value than that of cells on New-Ti (*p* < 0.01; Fig. 6a). H(−)L(+) and H(+)L(+) treatments significantly increased the MTT value of cells on *Aa* biofilm-Ti compared with that of H(−)L(−) and H(+)L(−) treatments (*p* < 0.01). H(+)L(+)-treated *Aa* biofilm-Ti also showed significantly increased MTT values compared with those on New-Ti and *Aa* biofilm-Ti treated with H(−)L(+) (*p* < 0.01). NR assays showed similar tendencies as the MTT assays (Fig. 6b); cells cultured for 3 d on H(+)L(+)-treated *Aa* biofilm-Ti showed significantly higher NR values than those on H(−)L(−), H(+)L(−) or H(−)L(+)-treated *Aa* biofilm-Ti (*p* < 0.05). Unlike the MTT assay results, there were no significant differences in NR value between New-Ti and *Aa* biofilm-Ti treated with H(+)L(+) (*p* = 0.07) and between New-Ti and *Aa* biofilm-Ti treated with H(−)L(−) (*p* > 0.05).

Regarding CHX and PI treatment, the MTT and NR values of cells on treated *Aa* biofilm-Ti were significantly lower than those of cells on New-Ti (*p* < 0.01) and were not significantly different from those of cells on *Aa* biofilm-Ti treated with pure water (*p* > 0.05; Fig. 6c,d). In addition, treatment duration influenced the effect of H(+)L(+) treatment on the proliferation of cells on *Aa* biofilm-Ti (Fig. 6e,f). The longer the treatment duration, the higher the MTT and NR values.

CLSM revealed that cell density on H(−)L(−)-treated *Aa* biofilm-Ti after 3-d culture was sparser than that on New-Ti, whereas cell density on H(+)L(+)-treated *Aa* biofilm-Ti was comparable to that on New-Ti (Fig. 6g). Quantitative analysis confirmed that the number of cells on H(−)L(−)-treated *Aa* biofilm-Ti was significantly lower than that of New-Ti and *Aa* biofilm-Ti treated with H(+)L(+) (*p* < 0.01); however, there were no significant differences between New-Ti and *Aa* biofilm-Ti treated with H(+)L(+) (*p* > 0.05; Fig. 6h). In addition, cell morphometric analysis revealed that cells attached to H(−)L(−)-treated *Aa* biofilm-Ti remained small after 3-h culture, whereas those attached to New-Ti and *Aa* biofilm-Ti treated with H(+)L(+) became elongated (Fig. S4a). Accordingly, the area, Feret’s diameter, and perimeter of cells grown on H(−)L(−)-treated *Aa* biofilm-Ti were significantly decreased compared with cells grown on New-Ti and *Aa* biofilm-Ti treated with H(+)L(+) (*p* < 0.05; Fig. S4b). Furthermore, the area dimensions of cells grown on H(+)L(+)-treated *Aa* biofilm-Ti were significantly higher than those grown on New-Ti (*p* < 0.01).

### Osteoblast proliferation on peri-implantitis-related three-species biofilm

**Figure 7:**
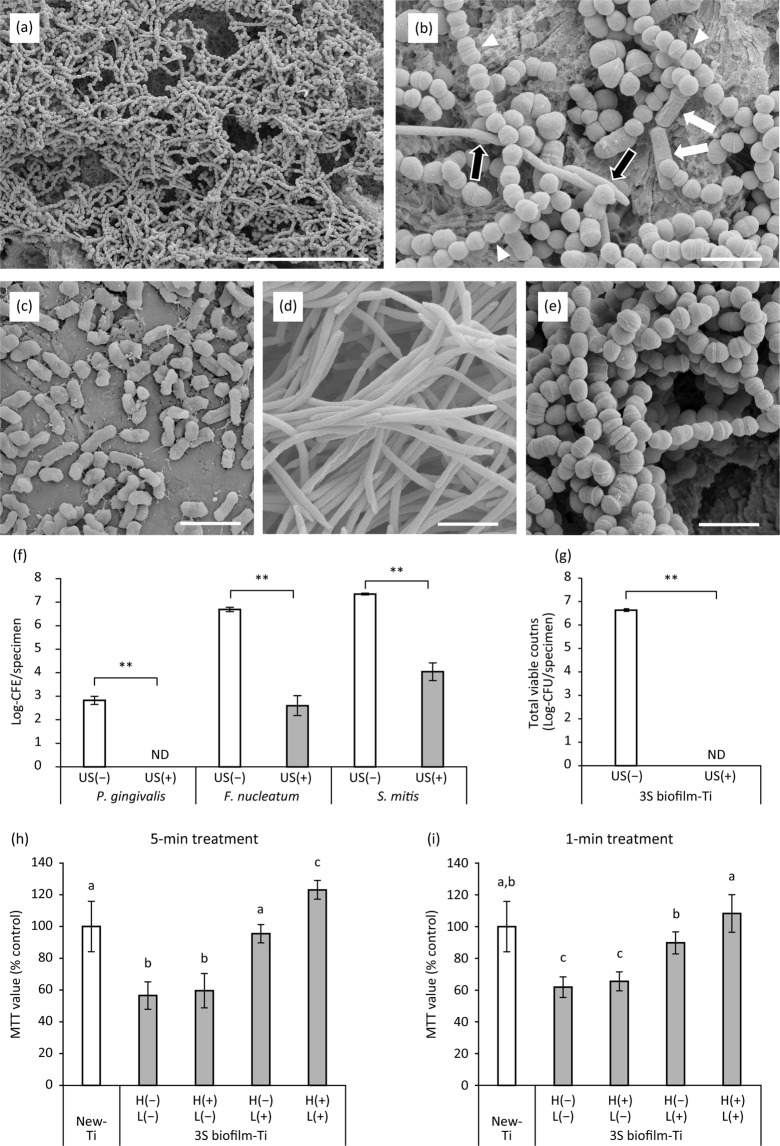
Characterisation of the three-species biofilm (3S biofilm) composed of *P*. *gingivalis*, *F*. *nucleatum*, and *S*. *mitis* as well as proliferation of MC3T3-E1 osteoblastic cells on 3S biofilm-contaminated titanium (3S biofilm-Ti) treated with H_2_O_2_ photolysis. (**a**,**b**) Representative scanning electron microscopy (SEM) images of 3S biofilm obtained at low and high magnification. (**b**) Based on morphology, *P*. *gingivalis*, *F*. *nucleatum*, and *S*. *mitis* are indicated by white arrows, black arrows, and white arrowheads, respectively. (**c**–**e**) Representative SEM images of (**c**) *P*. *gingivalis*, (**d**) *F*. *nucleatum*, and (**e**) *S*. *mitis*. Scale bar = 20 µm (**a**) and 2 µm (**b**–**e**). (**f**) Composition of the 3S biofilm with and without ultrasound scaling (US) analysed by qPCR. The values are expressed as logarithmic values of colony forming equivalents (CFE). (**g**) Total viable counts on 3S biofilm-Ti evaluated by the culture method. (**h**) Relative optical density values obtained via MTT assays (MTT value) for cells grown on 3S biofilm-Ti immersed in 3% H_2_O_2_ and irradiated with 365 nm LED, either alone or in combination denoted as H(−)L(−), H(+)L(−), H(−)L(+), or H(+)L(+), for 5 min. (**i**) Relative MTT values for cells grown on 3S biofilm-Ti under different treatments for 1 min. Regardless of treatment time, MTT values were significantly higher in H(+)L(+)-treated 3S biofilm-Ti than those treated with H(−)L(−), H(+)L(−), or H(−)L(+). Values and error bars in the graphs indicate the mean and standard deviation, respectively [n = 4 for (**f**,**g**) and n = 6 for (**h**,**i**)]. **Significant differences (*p* < 0.01) in CFE/specimen of each bacterial species or total viable counts between US(−) and US(+) groups in (**f**,**g**). Different letters above the columns in (**h**,**i**) refer to significant differences (*p* < 0.05) between different groups. ND, not detected. H(−)L(−), treatment with pure water in a light-shielding box; H(+)L(−), treatment with 3% H_2_O_2_ in a light-shielding box; H(−)L(+), 365-nm LED irradiation of sample in pure water; H(+)L(+), 365-nm LED irradiation of sample in 3% H_2_O_2_.

To verify the effect of H_2_O_2_ photolysis on cell proliferation under more clinically relevant conditions, a three-species biofilm (3S biofilm) composed of *Porphyromonas gingivalis*, *Fusobacterium nucleatum*, and *Streptococcus mitis* was prepared and used for cell proliferation assays. SEM images of the 3S biofilm and each bacterial species are shown in Fig. 7a–e. The entire surface of the titanium specimen was covered with many cocci followed by spindle-shaped rods and fewer short-rods, which correspond to *S*. *mitis*, *F*. *nucleatum*, and *P*. *gingivalis*, respectively. Quantitative real-time polymerase chain reaction (qPCR) analysis demonstrated that the colony forming equivalents (CFE) of *P*. *gingivalis*, *F*. *nucleatum*, and *S*. *mitis* in the 3S biofilm were 7.1 × 10^2^, 5.0 × 10^6^, and 2.2 × 10^7^ CFE/specimen, respectively (Fig. 7f). Total viable counts evaluated by the culture method were 4.4 × 10^6^ CFU/specimen (Fig. 7g), which was lower than the total of each bacterial CFE/specimen. Ultrasound scaling resulted in significant reduction of CFE/specimen for all three bacterial species (*P*. *gingivalis*, not detected; *F*. *nucleatum*, 6.0 × 10^2^ CFE/specimen; and *S*. *mitis*, 1.4 × 10^4^ CFE/specimen; Fig. 7f). Although bacterial DNA was detected by qPCR after ultrasound scaling, viable bacteria were not detected through culturing, regardless of bacterial species (Fig. 7g).

Proliferation of MC3T3-E1 cells cultured for 3 d on 3S biofilm-Ti showed similar tendencies to those cultured on *Aa* biofilm-Ti. In short, cells cultured on 5-min H(−)L(−)-treated 3S biofilm-Ti showed significantly lower MTT values than those cultured on New-Ti (*p* < 0.01; Fig. 7h). Five-minute H(−)L(+) and H(+)L(+) treatments significantly increased the MTT value of cells on 3S biofilm-Ti compared with that of H(−)L(−) and H(+)L(−) treatments (*p* < 0.01). Moreover, cells on 5-min H(+)L(+)-treated 3S biofilm-Ti showed significantly higher MTT values than those on New-Ti and 3S biofilm-Ti treated with H(−)L(+) (*p* < 0.01). When treatments were performed for 1 min, there were no significant differences in MTT value between cells on New-Ti and 3S biofilm-Ti treated with H(+)L(+) (*p* > 0.05), whereas H(+)L(+) resulted in significantly higher MTT values than did H(−)L(+) treatment (*p* < 0.01; Fig. 7i). Furthermore, H(−)L(−) and H(+)L(−) treatments resulted in significantly lower MTT values compared with that of other treatment groups and New-Ti (*p* < 0.01), consistent with observations for the 5-min treatments.

### Analysis of hydroxyl radical generation

**Figure 8:**
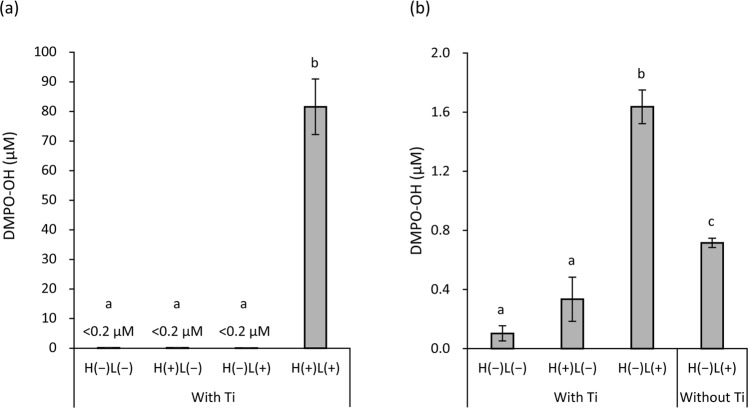
Quantification of hydroxyl radical spin adducts generated via LED irradiation of titanium specimens (Ti) immersed in 3% H_2_O_2_ or pure water. Analysis was performed using the electron spin resonance spin-trapping technique using 5,5-dimethyl-1-pyrroline *N*-oxide (DMPO) as a spin trap agent. The yield of DMPO-OH, a spin-adduct of hydroxyl radicals, (**a**) after treatment for 15 s and (**b**) 5 min. Irradiation with 365 nm LED of titanium specimens immersed in 3% H_2_O_2_ [H(+)L(+)] for 15 s generated 81.6 µM DMPO-OH, whereas other treatments generated only trace levels (<0.2 µM DMPO-OH). Values and error bars indicate the mean and standard deviation, respectively (n = 3). Different letters above the columns refer to significant differences (*p* < 0.01) between different groups. H(−)L(−), treatment with pure water in a light-shielding box; H(+)L(−), treatment with 3% H_2_O_2_ in a light-shielding box; H(−)L(+), 365-nm LED irradiation of sample in pure water; H(+)L(+), 365-nm LED irradiation of sample in 3% H_2_O_2_.

Electron spin resonance (ESR) spin-trapping analysis demonstrated that LED irradiation of titanium specimens immersed in 3% H_2_O_2_ for 15 s generated 81.6 µM 5,5-dimethyl-1-pyrroline *N*-oxide (DMPO)-OH, a spin-adduct of hydroxyl radicals, whereas other treatments generated only trace levels ( < 0.2 µM DMPO-OH; Fig. 8a). After extending the treatment duration to 5 min, H(−)L(−), H(+)L(−), and H(−)L(+) treatments resulted in 0.10, 0.33, and 1.64 µM DMPO-OH, respectively (Fig. 8b). H(−)L(+) treatment without titanium specimens yielded 0.72 µM DMPO-OH, which was significantly lower than that of H(−)L(+) treatment with titanium specimens (*p* < 0.01).

## Discussion

The present study showed that H_2_O_2_ photolysis can recover not only aged titanium surfaces but also biofilm-contaminated ones that cannot be completely cleaned by ultrasound scaling, thereby obtaining like-new conditions suitable for osteoblastic cell proliferation. Consequently, H_2_O_2_ photolysis enabled the MC3T3-E1 osteoblastic cells to proliferate on Aged-Ti, *Aa* biofilm-Ti, and 3S biofilm-Ti to the same extent as proliferation on New-Ti. Furthermore, this treatment did not affect the physical and chemical properties of intact titanium surfaces nor the associated cell response. Therefore, our results suggest that photolysis of 3% H_2_O_2_ with 365-nm LED irradiation can be utilised for chemical decontamination of aged and/or biofilm-contaminated titanium surfaces without material damage.

For clinically relevant findings, the effect of antimicrobial agents used for peri-implantitis treatment should be studied using titanium specimens with rough surfaces contaminated by microbial biofilms and the assessment of surface decontamination should involve quantification of residual biofilms^20^. The present study was performed in accordance with these recommendations. Titanium specimen surfaces were prepared to replicate the rough surfaces of dental implants, namely the sandblasted, large grit, and acid-etched (SLA) surfaces^27^. Analysis of surface roughness at the micro- and nano-scales confirmed that the specimens used herein had similar surface roughness to SLA surfaces^28^. Regarding surface contamination, *Aa* biofilms were used because clinical studies indicated the possible association of this bacterial species with peri-implantitis^29–32^. To mimic clinical procedures for implant surface debridement, ultrasound scaling using a PEEK tip was performed so as to not damage the rough surface of titanium in accordance with previous studies^33,34^, followed by treatment with each antimicrobial agent. Subsequently, biofilm-forming bacteria were quantified via colony enumeration assays after each treatment and the proliferation of osteoblastic cells on the treated titanium surface was evaluated. Cell proliferation was analysed via MTT and NR assays in the present study. MTT assays assess mitochondrial succinate dehydrogenase activity in viable cells^35^, whereas NR assays assess lysosomal incorporation of the dye in viable cells^36^. Although both measures are essentially correlated with the number of viable cells, they do not exactly reflect cell viability, especially when the function and/or number of mitochondria and lysosomes are affected by drug treatments^37^. Thus, both assays were used and the results were further confirmed via CLSM analysis.

The mechanism underlying ‘biological aging of titanium’ caused by storage under ambient conditions has been well documented by Att *et al*.^38^. They reported that aging is characterised by time-dependent accumulation of hydrocarbons on the titanium surface. Although newly prepared titanium surfaces are hydrophilic due to the affinity of the positively charged TiO_2_ layer at the outermost titanium surface for polar water molecules, accumulation of non-polar hydrocarbons makes the surface hydrophobic^39^. In addition, loss of the electropositive surface is not favourable for cellular attachment because cells and proteins associated with cell adhesion are negatively charged^39,40^. Att *et al*.^38–42^, who reported the aging of titanium, conducted extensive research on this issue, and devised a technique to recover the lost bioactivity of aged titanium surfaces, which was termed ‘photo-functionalization’. Photo-functionalization is performed using ultraviolet (UV) irradiation, which decreases the amount of hydrocarbons on the surface of aged titanium, converting the hydrophobic surface to a hydrophilic one. On the UV-treated surfaces of aged titanium, osteoblasts showed enhanced proliferation when compared with those on untreated surfaces of aged titanium. In the present study, H_2_O_2_ photolysis [H(+)L(+)] induced analogous chemical and biological changes. By contrast, LED irradiation at 365 nm without H_2_O_2_ [H(−)L(+)] did not significantly influence carbon reduction and cell proliferation on aged titanium surfaces, suggesting that H_2_O_2_ photolysis exerts its effect through a different mechanism than that of photo-functionalization. For photo-functionalization, aged titanium is generally irradiated with UVC (250 nm) at 2 mW/cm^2^ for 48 h^40–42^. Thus, the radiant exposure is calculated to be 345.6 J/cm^2^ (0.002 W/cm^2^ × 172,800 s). Herein, the specimens were irradiated with UVA (365 nm) at 1000 mW/cm^2^ for 5 min, corresponding to a radiant exposure of 300 J/cm^2^ (1 W/cm^2^ × 300 s). Although the radiant exposure provided was comparable, UVA irradiation did not result in photo-functionalization, suggesting that this effect depends on the wavelength of light, concurrent with previous reports wherein UVA treatment of aged titanium displayed no effects on osteoblast attachment and proliferation^41,42^. UVC irradiation has been suggested to eliminate carbon on titanium surfaces through two mechanisms, photocatalytic activity of the TiO_2_ layer and direct decomposition of carbon compounds by UVC^42^. UVA can also induce photocatalytic reactions with TiO_2_. The photon energy of UVA at 365 nm—calculated by the equation of *E* = *hc/λ*, where *E* is energy, *h* is Planck’s constant, *c* is the speed of light, and *λ* is the wavelength—is 3.4 eV, which is larger than the band gap of TiO_2_ (3.2 eV for anatase)^43^. Moreover, the 365-nm LED irradiation of titanium specimens immersed in pure water resulted in more than double the yield of hydroxyl radicals compared with irradiation of pure water without titanium specimens, as evidenced by ESR analysis and probably owing to the photocatalytic activity of TiO_2_. This finding suggests that hydroxyl radicals generated by UVA-induced photocatalysis are not sufficient for reducing carbon levels on aged titanium surfaces as shown in Fig. 2b. Therefore, H_2_O_2_ photolysis, which generates more hydroxyl radicals than photocatalysis, possibly results in the decomposition of accumulated carbon compounds on aged titanium surfaces.

When assessing cell proliferation on Aged-Ti, MTT and NR assays showed similar results regarding H(+)L(+) treatment in recovering the aging-induced reduction in cell proliferation; however, there were some discrepancies. For instance, although MTT assay results suggested that H(+)L(+) treatment accelerated cell proliferation on Aged-Ti, this proliferation was still much lower than cell proliferation on New-Ti. On the other hand, NR assays showed comparable cell viability between H(+)L(+)-treated Aged-Ti and New-Ti, whereas the other treatments resulted in significantly decreased cell viability compared with that of New-Ti. The discrepancy between MTT and NR assays probably results from differences in assay conditions, as described above. CLSM analysis demonstrated that the number of cells on H(+)L(+)-treated Aged-Ti was equivalent to that on New-Ti, whereas the number of cells on H(−)L(−)-treated Aged-Ti was approximately half in comparison. Considering this, it was determined that H_2_O_2_ photolysis can recover the surface condition of aged titanium for subsequent osteoblast proliferation to achieve the same conditions as newly prepared materials. However, the activity and number of mitochondria and/or lysosomes in osteoblastic cells growing on aged titanium surfaces treated with H_2_O_2_ photolysis may be affected via modified surface properties, resulting in the observed discrepancy between MTT and NR assays. We intend to further investigate whether this discrepancy affects the establishment of osseointegration in future studies.

Biofilm contamination assays confirmed that *Aa* biofilms on titanium surfaces cannot be detached from the surface by simply washing with saline. CLSM and SEM confirmed that the biofilm was comprised of bacterial cells and extracellular matrix components. The initial viable count of 1.3 × 10^5^ CFU/specimen in the biofilm was reduced to 1.5 × 10^2^ CFU/specimen when subjected to ultrasound scaling. Although ultrasound scaling effectively eliminated microbial biofilms, it could not completely eliminate bacteria, as evidenced by colony counting assays, CLSM, and SEM, which is concurrent with a previous report^33^. Moreover, SEM revealed that protrusions of micro-roughened titanium surfaces appeared collapsed in some parts after ultrasound scaling. In the backscattered electron image, a clear contrast was observed between the intact titanium surface and protrusions. Since the yield of backscattered electrons depends on the constituent element, the dark image at the protrusions indicates the presence of an element other than titanium with a lower atomic number. Thus, protrusions of micro-roughened titanium surfaces were plugged by remnants of the PEEK tip rather than being mechanically damaged. This was also confirmed via XPS wherein increased carbon compounds on New-Ti were observed after ultrasound scaling. Bacteria left on the titanium surface after ultrasound scaling were subjected to additional antimicrobial treatments. All antimicrobial treatments tested herein reduced the viable counts below the detection limit (2 CFU) after 5 min of treatment. Because viable bacterial cell counts were reduced via ultrasound scaling prior to antimicrobial treatment, differences in the bactericidal effect between treatments could not be detected. Therefore, cell proliferation assays following antimicrobial treatments may be barely affected by viable bacteria and solely influenced by alterations in surface conditions caused by biofilm contamination and treatments.

XPS demonstrated that ultrasound scaling of *Aa* biofilm-Ti yielded a higher atomic percentage of carbon than did New-Ti. Furthermore, *Aa* biofilm-Ti exhibited a nitrogen peak that was not present for New-Ti. These findings confirmed that biofilm contamination increased the percentage of both carbon and nitrogen on the titanium surface as reported previously^44^. Although H(+)L(−) treatment did not affect the percentage of carbon, H(−)L(+) and H(+)L(+) significantly decreased the percentage of carbon on *Aa* biofilm-Ti. The effect of H(−)L(+) is probably attributable to photocatalytic action of the TiO_2_ layer formed on specimen surfaces, although the same treatment did not significantly decrease the atomic percentage of carbon accumulated on Aged-Ti. This may be due to the differing affinities of different types of carbon compounds derived from biofilm contamination and aging. As H_2_O_2_ photolysis can more effectively reduce the carbon on *Aa* biofilm-Ti than can H(−)L(+), H_2_O_2_ photolysis can thus effectively reduce carbon compounds regardless of origin.

Furthermore, H_2_O_2_ photolysis was able to recondition *Aa* biofilm-Ti surfaces for subsequent osteoblastic cell proliferation. MTT assays revealed that cell proliferation on H(−)L(−)-treated *Aa* biofilm-Ti was decreased compared with that on New-Ti. Conversely, cell proliferation on H(+)L(+)-treated *Aa* biofilm-Ti increased compared with that on New-Ti. Furthermore, H(−)L(+)-treated *Aa* biofilm-Ti yielded comparable MTT assay results to that of New-Ti. Similar tendencies were observed for NR assays. Comparison of H(+)L(+) and H(−)L(+) suggests that the former reconditions titanium surfaces contaminated with microbial biofilms more effectively for subsequent osteoblastic cell proliferation than can the latter. Considering XPS and cell proliferation assay results, this accelerated cell proliferation is possibly associated with elimination of carbon contamination by each treatment. Additionally, the effect of H(+)L(+) was enhanced in a time-dependent manner. Of the conditions tested, 5-min H_2_O_2_ photolysis exerted the highest effect on cell proliferation. In contrast, treatment with antiseptics, such as 3% H_2_O_2_ alone, 0.5% PI, and 0.2% CHX, exerted minor effects on subsequent cell proliferation on *Aa* biofilm-Ti, although all treatments tested reduced viable bacterial counts to below the detection limit. CLSM also confirmed the H_2_O_2_ photolysis treatment effects observed in the MTT and NR assays. Osteoblastic cells attached to *Aa* biofilm-Ti and cultured for 3 h were less elongated compared with those on New-Ti. However, elongation of cells attached to H_2_O_2_ photolysis-treated *Aa* biofilm-Ti were comparable to or more than those on New-Ti after 3-h culture. Initial cell-biomaterial interactions, such as cell attachment and spreading, generally regulate subsequent function^45,46^. For instance, cell spreading enhances osteoblast differentiation and osteoblast matrix deposition^47,48^. Unsuccessful elongation of osteogenic cells may result in little induction of osteogenic phenotypes and may lead to their de-differentiation^40,49^. Thus, the cell spreading on H_2_O_2_ photolysis-treated *Aa* biofilm-Ti observed in the present study indicates that this treatment can re-establish the surface for favourable cell-biomaterial interactions. Additionally, after 3-d culture, the cell count on H_2_O_2_ photolysis-treated *Aa* biofilm-Ti was equivalent to that on New-Ti, whereas the number of cells on H(−)L(−)-treated *Aa* biofilm-Ti was significantly decreased compared with those on New-Ti and H_2_O_2_ photolysis-treated *Aa* biofilm-Ti.

To verify the findings obtained using the *Aa* biofilm model, 3S biofilm-Ti—a more clinically relevant model—was prepared using the peri-implantitis-related bacteria *P*. *gingivalis*, *F*. *nucleatum*, and *S*. *mitis*^50^ in basal mucin medium, which is frequently used as artificial saliva^51–53^, and subjected to MTT assays. Composition of the 3S biofilm was similar to a previous report^53^, whereby *S*. *mitis* was the most dominant species followed by *F*. *nucleatum* and then *P*. *gingivalis* in small amounts. The finding that total CFE/specimen was higher than total CFU/specimen indicates that the biofilm contained a substantial number of dead bacterial cells. Although ultrasound scaling resulted in no detection of viable bacteria, the titanium surface still contained dead bacterial cells and/or extracellular bacterial DNA as demonstrated by qPCR. Thus, as in the case of the *Aa* biofilm, the reduced cell proliferation on H(−)L(−)-treated 3S biofilm-Ti compared with New-Ti is possibly due to contaminants such as hydrocarbons derived from biofilm remnants, including the dead bacterial cells. In line with the *Aa* biofilm model, 5-min H(+)L(+) and H(−)L(+) treatments recovered cell proliferation on the 3S biofilm-Ti to comparable or higher levels than that on New-Ti, whereas H(+)L(−) exerted only minor effects. Again, comparing H(+)L(+) and H(−)L(+) suggested that the former reconditions titanium surfaces contaminated with biofilms more effectively for subsequent osteoblastic cell proliferation than the latter in relation to hydroxyl radical yield. In addition, it was demonstrated that even 1-min H(+)L(+) treatments recovered cell proliferation to comparable levels as on New-Ti, indicating that treatment time may be shortened to less than 5 min. Considering the cell proliferation assay results of both *Aa* and 3S biofilm models, osteoblastic cells are able to proliferate on biofilm-contaminated titanium surfaces after H_2_O_2_ photolysis treatment and cell proliferation can be recovered to the same levels as on New-Ti.

Nevertheless, the present study has some limitations. For instance, only biofilm models composed of limited bacterial species were used to simplify the study for evaluation of various testing conditions, even though clinical biofilms at peri-implantitis sites have a much larger diversity composed of >150 microbial species, as demonstrated by recent metagenomics studies^54,55^. In addition, it should be noted that although they are associated with periodontitis, the bacterial species used in the present study, especially *A*. *actinomycetemcomitans*, may not represent peri-implantitis pathogens as the microbiomes of periodontal and peri-implant biofilm microbiomes may be distinct^56–58^. Accordingly, the findings of the present study should be validated using more clinically relevant models, such as an *ex vivo* biofilm model (using microorganisms collected from peri-implantitis sites) or an *in vivo* peri-implantitis model. Another limitation is that the mouse osteoblastic cell line was used instead of cells originating from humans, and bone formation on biofilm-contaminated titanium surfaces was not evaluated. Therefore, more extensive studies are required to verify efficacy of the H_2_O_2_ photolysis technique in peri-implantitis treatment.

Within the limitations of the present study, we concluded that H_2_O_2_ photolysis can eliminate accumulated carbon compounds on titanium surfaces due to both aging and microbial biofilm contamination as well as enhance subsequent osteoblastic cell proliferation. In contrast, conventional antiseptics, such as PI, CHX, and H_2_O_2_ (without LED irradiation), did not recover cell proliferation on biofilm-contaminated titanium surfaces. Although the effects of H_2_O_2_ photolysis and photo-functionalization are similar, it may be difficult to apply the latter treatment to peri-implantitis because UVC irradiation can damage the DNA of host tissue cells^59^. As for the safety of H_2_O_2_ photolysis, 3% H_2_O_2_ is regarded as acceptable^60^ and UVA has also been employed in photo-therapy for the treatment of skin conditions^61,62^. In addition, exposure of the oral mucosa to hydroxyl radicals for a short period of time does not cause abnormal changes, as reported in previous *in vivo* studies^63,64^. Although safety assessment of this treatment—5-min UVA irradiation of 3% H_2_O_2_ on the lesion site—should be conducted before clinical application, H_2_O_2_ photolysis has potential for use as a peri-implantitis treatment by inactivating microbial biofilms and reconditioning titanium surfaces for subsequent osteoblast proliferation with regard to efficacy.

## Materials and Methods

### Preparation of titanium specimens

Commercially pure titanium discs (Grade 4; TB550; Nishimura, Fukui, Japan) with 5-mm diameters and 2-mm thick were sandblasted and acid-etched to obtain a rough surface similar to that of commercial dental implants, namely SLA surfaces. Sandblasting was performed using 250-µm alumina particles at a blasting pressure of 0.4 MPa. The specimens were then acid etched in 49% (v/v) sulfuric acid for 1 h at 60 °C and cleaned via ultrasonic cleaning in ultrapure water (10 min), acetone (10 min), and again in ultrapure water (10 min). After cleaning, the specimens were autoclaved for 15 min at 121 °C. Acid etching, cleaning, and autoclaving were performed the day before each assay, unless otherwise specified.

### Assessment of H_2_O_2_ photolysis on titanium surfaces without biofilm contamination

Titanium discs were immersed in 3% (w/v) H_2_O_2_ and irradiated with LED for 5 min, either alone or in combination and denoted H(−)L(−), H(+)L(−), H(−)L(+), or H(+)L(+). H(+) specimens were immersed in 300 µL 3% (w/v) H_2_O_2_ (Santoku Chemical, Tokyo, Japan), whereas H(−) specimens were immersed in 300 µL ultrapure water. Analogously, L(+) specimens were irradiated with 365-nm LED light at 1000 mW/cm^2^, whereas L(−) specimens were maintained in a light-shielding box. We used an LED spot-curing device (OmniCure LX400; Lumen Dynamics Group, Mississauga, Canada) as the light source. The specimens were washed twice with ultrapure water and assayed.

Surface topography of newly prepared titanium discs (New-Ti) treated with H(−)L(−) and H(+)L(+) was examined using an SEM (JSM-7100F; JEOL, Tokyo, Japan), an optical interferometer (Talysurf CCI HD; Taylor Hobson, Leicester, UK), and an AFM (SFT-3500; Shimadzu, Kyoto, Japan). The SEM was operated at 5 kV, the optical interferometer under a 50 × objective lens to analyse surface roughness at the micro-scale, and the AFM in tapping mode using a silicon cantilever (spring constant, 2 N/m; resonant frequency, 70 kHz) and with a scan range of 1 × 1 µm for analysing surface roughness at the nano-scale. Six independent assays were performed for each test.

The crystalline structures of New-Ti treated with H(−)L(−) or H(+)L(+) were analysed using a *θ*–2*θ* XRD (SmartLab; Rigaku, Tokyo, Japan). Diffractograms were obtained from 20° to 65° at a scan speed of 10°/min and a step size of 0.01° using Cu-Kα radiation. A high-sensitive 2D detector (HyPix-400; Rigaku) was used to detect a thin layer of TiO_2_ (i.e. passive film). Diffractogram peaks were qualified using standard models recorded in the International Centre for Diffraction Data and TiO_2_ peak intensities were computed using the software package PDXL (Rigaku). Four independent assays were performed for each test.

Surface chemical composition was examined using an XPS (JPS-9010MC; JEOL). New-Ti as well as titanium discs aseptically stored in an incubator at 37 °C for 4 weeks (Aged-Ti) were included in this analysis. New-Ti treated with H(−)L(−) or H(+)L(+) and Aged-Ti treated with H(−)L(−), H(+)L(−), H(−)L(+), or H(+)L(+) were subjected to XPS. The XPS device was operated at 10 kV and 10 mA as well as monochromatic Mg-Kα X-ray radiation with a spot size of 1 mm. The results were evaluated using the software package SpecXPS (JEOL) and four independent assays were performed for each test.

The wettability of New-Ti and Aged-Ti treated with H(−)L(−) and H(+)L(+) was evaluated using a contact angle meter (CA-X, Kyowa Interface Science, Saitama, Japan). The contact angle of 0.4 µL ultrapure water was measured and six independent assays were performed for each test.

### A.actinomycetemcomitans biofilm contamination

The test strain *A*. *actinomycetemcomitans* JCM 2434 was obtained from the Japan Collection of Microorganisms (RIKEN BioResource Center, Wako, Japan). The study design is illustrated in Fig. S5 and biofilm formation was induced using a previously reported method^65^. The single-species biofilm model, instead of a multi-species biofilm, was used as a simplified model to evaluate various testing conditions as described below. Bacteria were grown anaerobically using AneroPack (Mitsubishi Gas Chemical Company, Tokyo, Japan) in brain heart infusion (BHI) broth (Becton Dickinson, Franklin Lakes, NJ) supplemented with 10 g/L yeast extract (Oxoid, Hampshire, UK) at 37 °C for 24 h. From this culture, a bacterial suspension was prepared in sterile saline and the concentration was adjusted to approximately 5 × 10^8^ CFU/mL. Autoclaved titanium discs were placed in 48-well plates, and then 1 mL BHI broth supplemented with 10 g/L yeast extract and 100 µL bacterial suspension (inoculum = 5 × 10^7^ CFU) were added to the wells, after which the plates were incubated under anaerobic conditions at 37 °C for 48 h. Thereafter, the specimens were gently washed twice with saline to eliminate non-attached bacteria and used in subsequent assays as *Aa* biofilm-Ti.

### Assessment of ultrasound scaling followed by H_2_O_2_ photolysis on *A*. *actinomycetemcomitans* biofilm-contaminated titanium

*Aa* biofilm-Ti was subjected to ultrasound scaling (mechanical removal of biofilm), followed by each antimicrobial treatment to mimic the clinical procedure of peri-implantitis treatment. Ultrasound scaling was performed using a power-driven scaler (miniMaster Piezon LED, EMS, Nyon, Switzerland) with a plastic scaler tip made of PEEK (ITMS Peek tip straight, Star Chip, Himeji, Japan). Instrument power was set to 70% and pure water at a flow rate of 50 mL/min was used as water coolant. The titanium specimen was held using forceps and the entire biofilm-containing surface was subjected to ultrasound scaling for 1 min. After ultrasound scaling, the specimens were treated with either H(−)L(−), H(+)L(−), H(−)L(+), H(+)L(+), 0.2% (w/v) CHX or 0.5% (w/v) PI for 5 min. H(−)L(−), H(+)L(−), H(−)L(+), and H(+)L(+) were performed as described above. CHX and PI were prepared by diluting 20% (w/v) CHX solution (Toyo Seiyaku Kasei, Osaka, Japan) and 7% (w/v) PI solution (Meiji Seika Pharma, Tokyo, Japan) in pure water and then *Aa* biofilm-Ti specimens were immersed in 300 µL of each antiseptic under a light-shielded condition.

*Aa* biofilm-Ti specimens subjected to each treatment were washed twice with saline to stop the reactions. Thereafter, the remaining bacteria in the biofilm were harvested via the enzymatic detachment method as previously described but with some modifications^66^. Each treated *Aa* biofilm-Ti was immersed in 300 µL enzyme suspension composed of 4 mg/mL type I collagenase (Thermo Fisher Scientific, Waltham, MA) and 2 mg/mL dispase (Thermo Fisher Scientific) in phosphate-buffered saline (PBS). The 48-well plate containing samples was incubated for 2 h under rotation at 200 rpm and 37 °C. Subsequently, the sample and enzyme solution were transferred to 1.5-mL micro-tubes and vortexed for 10 s. The suspension was serially diluted 10-fold in saline and then 150 µL of the dilution was plated onto BHI agar (Becton Dickinson). Agar plates were cultured anaerobically at 37 °C for 48 h, followed by colony counting to determine CFU/specimen. Viable bacterial counts for *Aa* biofilm-Ti without any treatments were also evaluated. Three independent assays were performed for each test.

*Aa* biofilm-Ti with or without ultrasound scaling was analysed by CLSM and SEM. For CLSM, the biofilm samples were fluorescently stained with 10 µM SYTO9 (Thermo Fisher Scientific) for 20 min at room temperature (20–25 °C). Thereafter, samples immersed in pure water were imaged using a laser at an excitation wavelength of 488 nm with a CLSM (TCS-SPE; Leica, Wetzlar, Germany). For SEM, *Aa* biofilm-Ti was fixed with 2.5% glutaraldehyde, post-fixed with 2% osmium tetroxide, dehydrated through a graded ethanol series, and freeze-dried in *t*-butyl alcohol. Each freeze-dried sample was coated with platinum and then secondary electron images of the sample were obtained using an SEM (JSM-7100F) operated at 5 kV. In addition, backscattered electron images were also obtained using the SEM operated at 15 kV to examine the presence of PEEK tip remnants on the titanium surface after ultrasound scaling.

The surface chemical composition of New-Ti treated with or without ultrasound scaling and *Aa* biofilm-Ti treated with ultrasound scaling followed by H(−)L(−), H(+)L(−), H(−)L(+), or H(+)L(+) was examined using an XPS under the same aforementioned conditions. Three independent assays were performed for each test.

### Cell culture and cell proliferation assays with the *A*. *actinomycetemcomitans* biofilm model

The mouse osteoblastic cell line MC3T3-E1 was obtained from the RIKEN Cell Bank (Tsukuba, Japan). Cells were sub-cultured in a cell culture flask with alpha-modified Eagle’s medium (α-MEM; Nacalai Tesque, Kyoto, Japan) supplemented with 10% (v/v) foetal bovine serum (FBS; Thermo Fisher Scientific), 100 U/mL penicillin, and 0.1 mg/mL streptomycin (P/S; Wako Pure Chemicals Industries, Osaka, Japan). At 80% confluence, the cells were detached using 0.25% (w/v) trypsin-EDTA (Thermo Fisher Scientific) and re-suspended in an osteogenic medium [α-MEM supplemented with 10% (v/v) FBS, P/S, 50 µg/mL ascorbic acid (Wako Pure Chemicals Industries), 10 mM β-glycerophosphate disodium (Sigma-Aldrich, St. Louis, MO), and 0.01 µM dexamethasone (Wako Pure Chemicals Industries)]^67^. Cell suspensions (500-µL aliquots) were then seeded onto each of the treated titanium specimens in 48-well plates at a density of 30,000 cells/well for cell proliferation assays and 3,000 cells/well for cell morphometric analysis. The plates were incubated at 37 °C in humidified 5% CO_2_ for 3 h or 3 d.

Cell proliferation was assessed via the MTT and NR assays and CLSM. For the MTT and NR assays, cell proliferation on New-Ti and Aged-Ti treated with H(−)L(−), H(+)L(−), H(−)L(+), or H(+)L(+) was examined. In addition, *Aa* biofilm-Ti treated with ultrasound scaling followed by H(−)L(−), H(+)L(−), H(−)L(+), H(+)L(+), CHX, or PI treatment were also examined. Furthermore, to evaluate the effect of treatment duration, *Aa* biofilm-Ti subjected to ultrasound scaling followed by H(+)L(+) treatment for 0, 1, 3, and 5 min was also subjected to the assays. For the MTT assay, MTT (Tokyo Chemical Industry, Tokyo, Japan) was dissolved in PBS and diluted in the medium for a final 0.1% (w/v) solution. The titanium specimens on which cells proliferated for 3 d (as described above) were immersed in 300 µL MTT solution and incubated for 2 h. Thereafter, MTT that was converted into insoluble formazan was solubilised using dimethyl sulfoxide and evaluated through colourimetric determination at 595 nm with a microplate reader (FilterMax F5; Molecular Devices, Sunnyvale, CA). For the NR assay, the titanium specimens were immersed in 300 µL of medium containing 150 µg/mL NR (Wako Pure Chemicals) and incubated for 3 h. Thereafter, NR incorporated into the cells was extracted with 50% (v/v) ethanol containing 1% (v/v) acetic acid for colourimetric determination at 540 nm using the microplate reader. Six independent assays were performed for each test.

For CLSM, cells grown on New-Ti, Aged-Ti treated with H(−)L(−) or H(+)L(+), and *Aa* biofilm-Ti treated with H(−)L(−) or H(+)L(+) were fixed in 4% paraformaldehyde and fluorescently stained. Nuclei and actin filaments were stained with 300 nM 4’,6-diamidino-2-phenylindole (DAPI; Thermo Fisher Scientific) and 165 nM rhodamine phalloidin (Thermo Fisher Scientific), respectively. To quantify the cells on *Aa* biofilm-Ti, nuclei were stained with 10 µM SYTO9 instead of DAPI as the latter increases background noise. The cells were imaged using the CLSM (TCS-SPE) at an excitation wavelength of 405 nm for DAPI, 488 nm for SYTO9, and 532 nm for rhodamine phalloidin. The obtained CLSM images were analysed using ImageJ (National Institute of Health, Bethesda, MD). Cells were quantified over nine CLSM images while cell morphometry was performed across 15 images.

### Preparation and characterisation of the three-species biofilm

The test strains *P*. *gingivalis* JCM 12257, *F*. *nucleatum* JCM 12990, and *S*. *mitis* JCM12971 were obtained from the Japan Collection of Microorganisms (RIKEN BioResource Center). *P*. *gingivalis* and *F*. *nucleatum* were grown anaerobically in BHI broth supplemented with 5 mg/L hemin (Tokyo Chemical Industry) and 1 mg/L menadione (Tokyo Chemical Industry) at 37 °C for 2 d. *S*. *mitis* was anaerobically grown in BHI broth for 20 h. From these cultures, bacterial suspensions of *P*. *gingivalis* and *S*. *mitis* were prepared in sterile saline while *F*. *nucleatum* was prepared in liquid Amies transport medium [3 g/L sodium chloride (Wako Pure Chemicals Industries), 1.15 g/L sodium hydrogen phosphate (Nacalai Tesque), 1 g/L sodium thioglycollate (Nacalai Tesque), 0.2 g/L potassium dihydrogen phosphate (Wako Pure Chemicals Industries), and 0.2 g/L potassium chloride (Wako Pure Chemicals Industries) in pure water]. The concentration of each bacterial suspension was adjusted to approximately 5 × 10^8^ CFU/mL. For biofilm formation, basal mucin medium^51–53^ was used which contains 2.5 g/L mucin from porcine stomach (Wako Pure Chemicals Industries), 3.5 g/L sodium chloride, 0.2 g/L potassium chloride, 0.2 g/L calcium chloride dihydrate (Wako Pure Chemicals Industries), 2 g/L yeast extract, 1 g/L lab-lemco powder (Oxoid), 5 g/L proteose peptone (Oxoid), 5 mg/L hemin, and 1 mg/L menadione in pure water. After autoclaving, urea (Wako Pure Chemicals Industries) was added for a final 0.5 g/L concentration. The autoclaved titanium discs were placed in 48-well plates, and then 820 µL basal mucin medium and 60 µL of each bacterial suspension (inoculum = 3 × 10^7^ CFU) were added to each well. Subsequently, the plates were incubated under anaerobic conditions at 37 °C for 48 h. Thereafter, the specimens were gently washed twice with saline to eliminate non-attached bacteria and used in the following assays as 3S biofilm-Ti.

3S biofilm-Ti as well as single species biofilms composed of each bacterial species were subjected to SEM analysis as described above. Furthermore, 3S biofilm composition with and without ultrasound scaling was analysed using qPCR. The biofilm-forming bacteria were detached by the aforementioned enzymatic method. Subsequently, bacterial DNA was extracted using PureLink Microbiome DNA Purification Kit (Thermo Fisher Scientific). Extracted DNA (2 µL) was added to a master mix of 10 µL SYBR Green (SSoAdvanced Universal SYBR Green Supermix, Bio-Rad Laboratories, Hercules, CA), 2 µL each of 5 µM forward and reverse primers specific for each bacterial species^68–70^ (Table S2), and 4 µL nuclease-free water. The reaction mixture was then subjected to qPCR using the MyiQ Single-Color Real-Time PCR Detection System (Bio-Rad Laboratories). To construct standard curves for each bacterial species, suspensions prepared in a 10^3^–10^8^ CFU/mL range were also subjected to qPCR. Based on the standard curves, CFE/specimen was calculated. In addition, total viable counts for 3S biofilms were evaluated by the culture method. Suspensions of detached biofilm-forming bacteria were serially diluted 10-fold in saline and then 50 µL of the dilution was plated onto Brucella HK agar (Kyokuto Pharmaceutical Industry, Tokyo, Japan) supplemented with 5% (v/v) defibrinated horse blood (Nippon Bio-Supp. Center, Tokyo, Japan). Agar plates were cultured anaerobically at 37 °C for 7 d, followed by colony counting for determination of CFU/specimen. Viable bacterial counts for 3S biofilm-Ti subjected to ultrasound scaling were also evaluated. Four independent assays were performed for each test.

### Cell proliferation assay using the three-species biofilm model

Proliferation of MC3T3-E1 cells cultured for 3 d on New-Ti and 3S biofilm-Ti treated with H(−)L(−), H(+)L(−), H(−)L(+), or H(+)L(+) was examined via MTT assays as described above. Each treatment was performed for 1 or 5 min and six independent assays were performed for each test.

### Analysis of hydroxyl radical generation

The hydroxyl radical yield generated by H(−)L(−), H(+)L(−), H(−)L(+), and H(+)L(+) treatments was analysed via ESR spin-trapping in accordance with a previous study^71^. Briefly, 300 µL of each solution was prepared for treatments [H(−)L(−), H(+)L(−), H(−)L(+), and H(+)L(+)] containing 300 mM DMPO (Labotec, Tokyo, Japan). Titanium specimens were immersed in the prepared solutions and irradiated with LED or maintained in a light-shielding box. Each treatment was performed for 15 s. H(−)L(−), H(+)L(−), and H(−)L(+) treatments generated only trace levels of hydroxyl radicals, and thus the treatment duration was extended to 5 min. Furthermore, the yield of hydroxyl radicals generated by H(−)L(+) treatment with or without titanium specimens was compared to estimate the photocatalytic activity of TiO_2_ formed on the titanium surface. ESR spectra of the samples were recorded using an X-band ESR spectrometer (JES-FA-100; JEOL). The concentration of hydroxyl radicals trapped by DMPO (DMPO-OH) was determined using the software package Digital Data Processing (JEOL). Three independent assays were performed for each test.

### Statistical analysis

Differences in surface roughness, atomic percentages of carbon, contact angles, bacterial cell count, cell proliferation parameters, and hydroxyl radical yield were assessed using the Student *t*-test for pairwise comparisons or analysis of variance (ANOVA) followed by the post-hoc Tukey-Kramer Honestly Significant Difference test for multiple comparisons. Statistical analyses were performed using JMP Pro 13.0 software (SAS Institute, Cary, NC). *p* values < 0.05 were considered statistically significant.

## Supplementary information

Supplementary Information

## Data Availability

The datasets generated and/or analysed during the current study are available from the corresponding author upon reasonable request.
