# Supplementary material for: Hydroxyl radicals generated by hydrogen peroxide photolysis recondition biofilm-contaminated titanium surfaces for subsequent osteoblastic cell proliferation
Source: Sci Rep. 2019 Mar 18;9:4688. doi: 10.1038/s41598-019-41126-z (PMC6423011; doi:10.1038/s41598-019-41126-z)
Supplement: Supplementary file 1 — Supplementary Information [file 41598_2019_41126_MOESM1_ESM.pdf]

Supplementary Information

Hydroxyl radicals generated by hydrogen peroxide photolysis recondition biofilm-contaminated titanium surfaces for subsequent osteoblastic cell proliferation

Keisuke Nakamura[1*], Midori Shirato[1], Taichi Tenkumo[1], Taro Kanno[1], Anna Westerlund[2], Ulf Örtengren[3,4], Keiichi Sasaki[1,5], and Yoshimi Niwano[6]

[1]Department of Advanced Free Radical Science, Tohoku University Graduate School of Dentistry, 4-1 Seiryo, Aoba-ku, Sendai 980-8575, Japan.

[2]Department of Orthodontics, Institute of Odontology, Sahlgrenska Academy, University of Gothenburg, Gothenburg 40530, Sweden.

[3]Department of Clinical Dentistry/Faculty of Health Sciences, The Arctic University of Norway, Tromsø 9037, Norway.

[4]Department of Cariology, Institute of Odontology, Sahlgrenska Academy, University of Gothenburg, Gothenburg 40530, Sweden.

[5]Division of Advanced Prosthetic Dentistry, Tohoku University Graduate school of Dentistry, 4-1 Seiryo, Aoba-ku, Sendai, 980-8575, Japan.

[6]Faculty of Nursing, Shumei University, 1-1 Daigaku-cho, Yachiyo, Chiba 276-0003, Japan

*keisuke@m.tohoku.ac.jp

Table S1. Viable bacterial counts on *A. actinomycetemcomitans* biofilm after each treatment

**Treatment**	**Log CFU/specimen**	
	**Ave**	**SD**
Untreated	5.12	0.30
US+H−L−	2.16	0.15
US+H+L−	ND	−
US+H−L+	ND	−
US+H+L+	ND	−
US+PI	ND	−
US+CHX	ND	−

US, 1-min ultrasound scaling; H, 3 % (w/v) H_2O_2; L, LED irradiation at 365 nm; PI, 0.5% (w/v) povidone-iodine; CHX, 0.2% (w/v) chlorhexidine gluconate; Ave, average; SD, standard deviation; ND, not detected.

Table S2. Primers used for qPCR in this study

**Bacterial species**		**Oligonucleotide sequence**	**Reference**
P. gingivalis	F	5'-ACCTTACCCGGGATTGAAATG-3'	68
	R	5'-CAACCATGCAGCACCTACATAGAA-3'	
F. nucleatum	F	5'-CTTAGGAATGAGACAGAGATG-3'	69
	R	5'-TGATGGTAACATACGAAAGG-3'	
Streptococcus spp. (*S. mitis*)	F	5'-GATACATAGCCGACCTGAG-3'	70
	R	5'-CCATTGCCGAAGATTCC-3'	

F, forward; R, reverse.

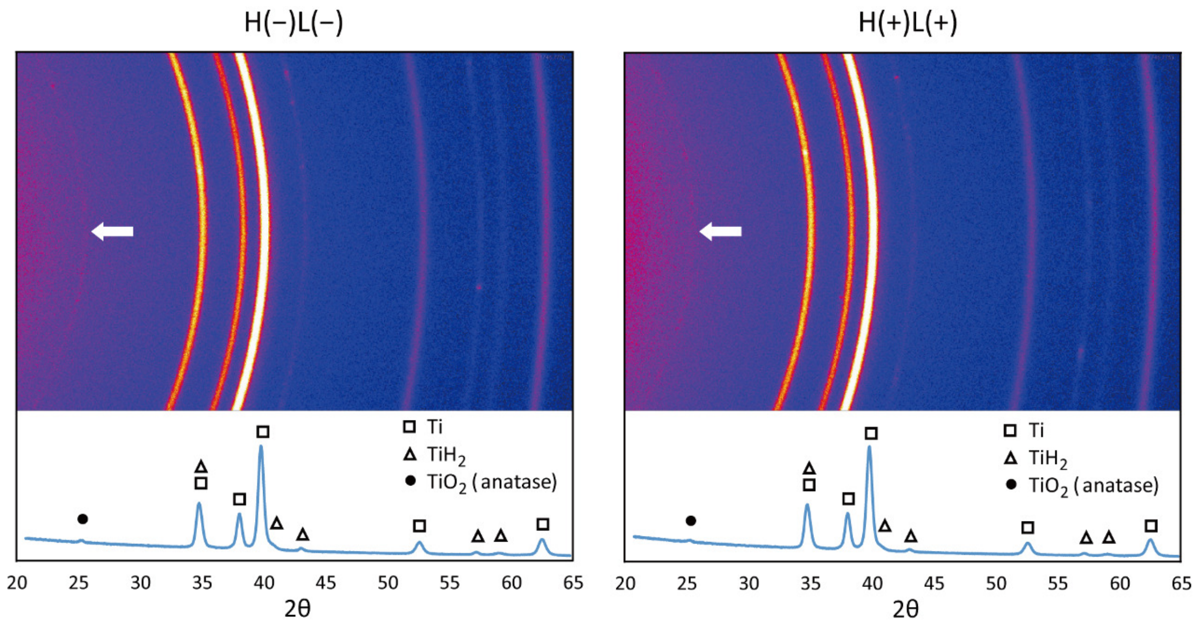

Figure S1. Representative 2D diffraction images and diffractograms of titanium specimens treated with or without H_2O_2 photolysis. Crystalline structures of treated New-Ti specimens were analysed using a θ–2θ X-ray diffractometer. H(−)L(−), treatment with ultrapure water; and H(+)L(+), treatment with H_2O_2 photolysis. White arrows indicate the Debye cone of TiO_2.

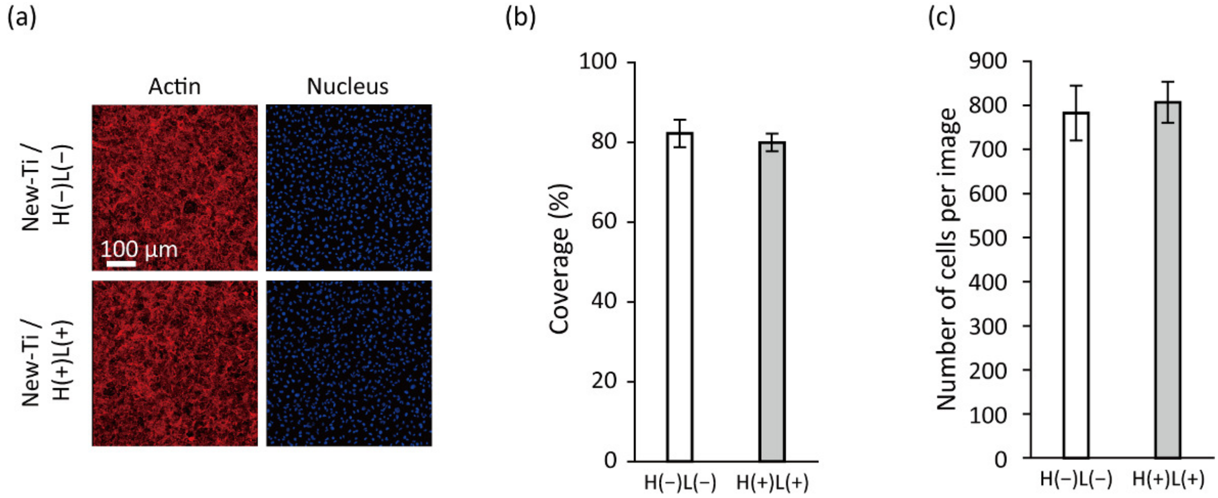

Figure S2. Confocal laser scanning microscopy of MC3T3-E1 osteoblastic cells cultured on titanium specimens. Cell nuclei and actin filaments were stained with 4’,6-diamidino-2-phenylindole (DAPI) and rhodamine phalloidin, respectively. (a) Representative images of cells cultured on New-Ti treated with H(−)L(−) and H(+)L(+) for 3 d. (b) Quantitative results of cellular coverage and (c) number of cells. Values and error bars in (b) and (c) indicate the mean and standard deviation, respectively (n = 9); statistical analysis revealed no differences between the groups. H(−)L(−): treatment with ultrapure water in a light-shielding box, H(+)L(+): LED irradiation of sample in 3% H_2O_2.

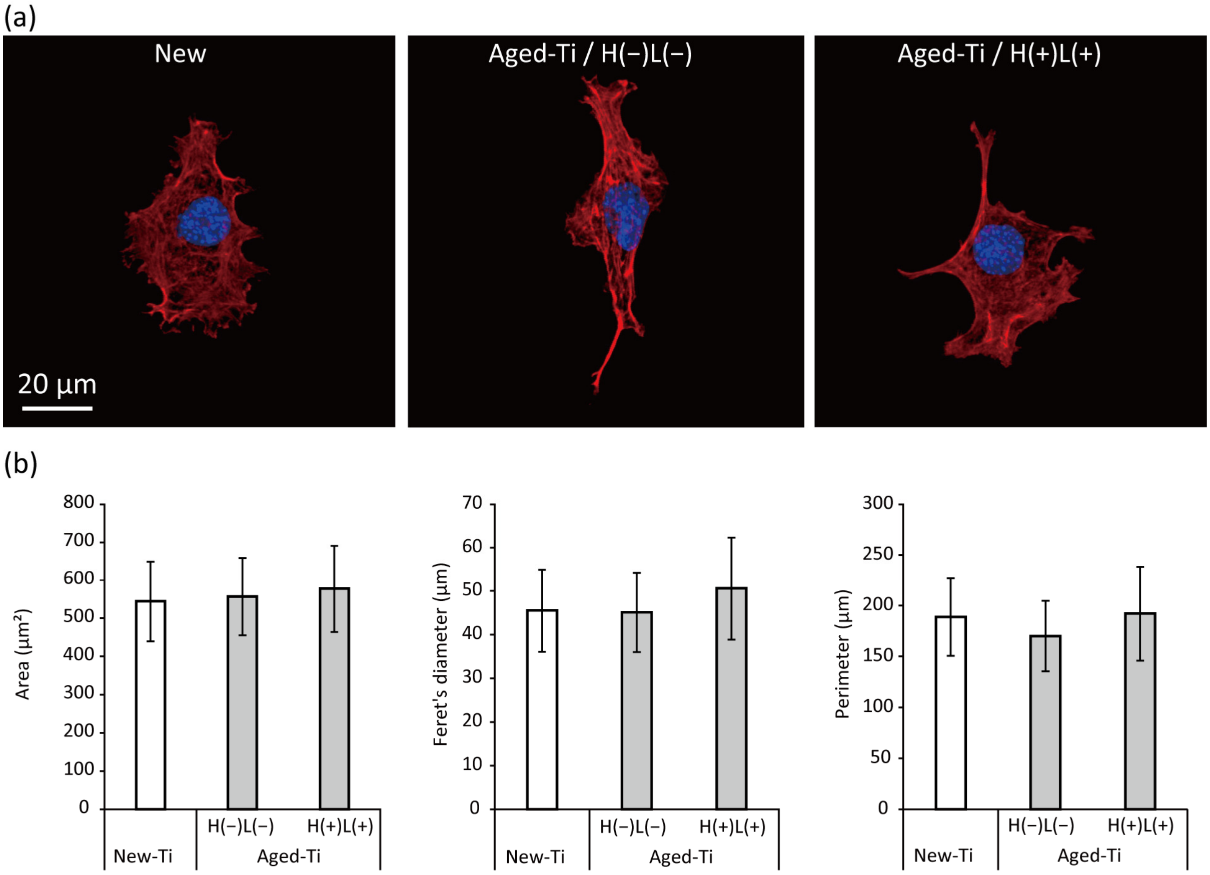

Figure S3. Morphometric analysis of MC3T3-E1 osteoblastic cells via confocal laser scanning microscopy. (a) Representative images of cells cultured for 3 h on aged titanium (Aged-Ti) treated with H_2O_2 photolysis. Nuclei and actin filaments were stained with 4',6-diamidino-2-phenylindole (DAPI) and rhodamine phalloidin, respectively. (b) Analysis of area, Feret's diameter and perimeter of the cells after 3 h of culturing. There were no significant differences ($p > 0.05$) in morphometric parameters between different groups. H(−)L(−), treatment with pure water in a light-shielding box; H(+)L(+), 365-nm LED irradiation of sample in 3% H_2O_2.

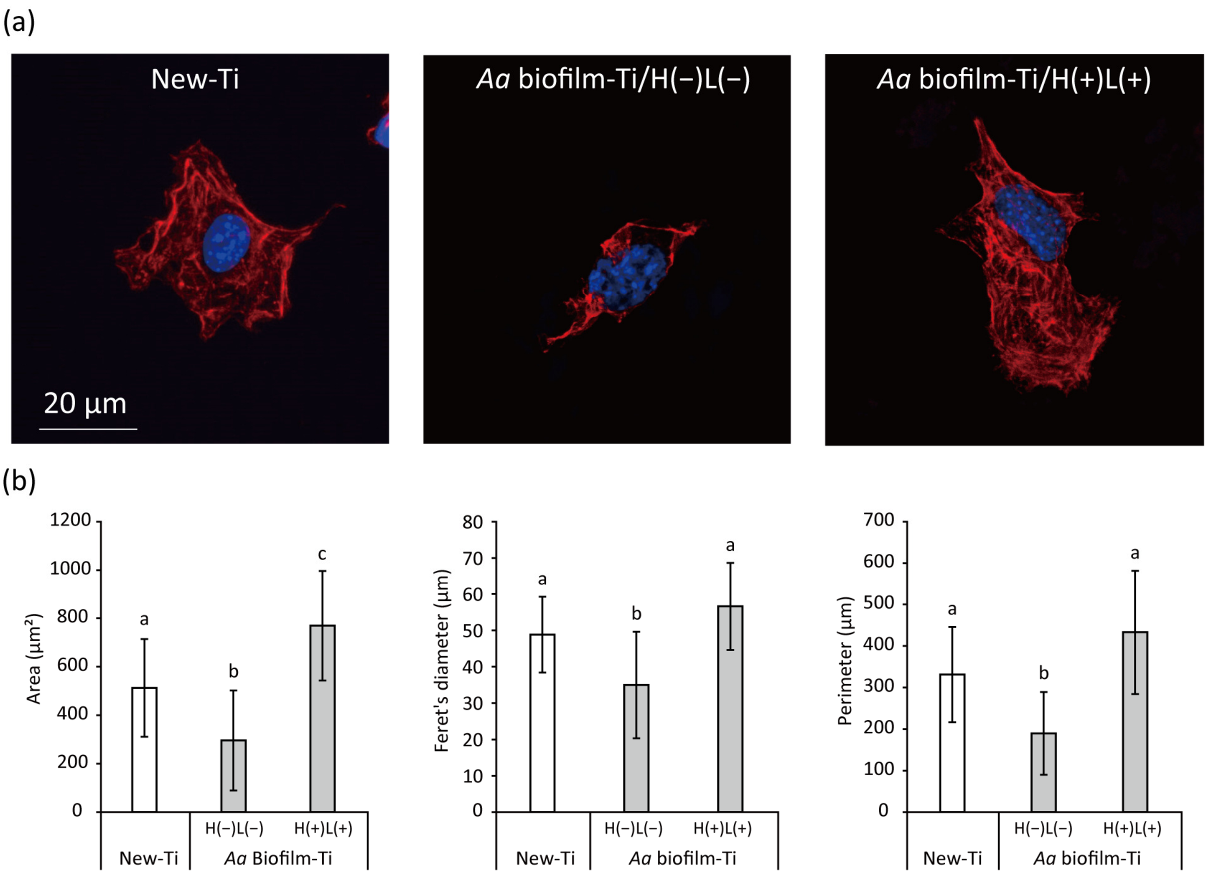

Figure S4. Morphometric analysis of MC3T3-E1 osteoblastic cells via confocal laser scanning microscopy. (a) Representative images of cells cultured for 3 h on *A. actinomycetemcomitans* biofilm-contaminated titanium (*Aa* biofilm-Ti) treated with H_2O_2 photolysis. Nuclei and actin filaments were stained with 4',6-diamidino-2-phenylindole (DAPI) and rhodamine phalloidin, respectively. (b) Analysis of area, Feret's diameter and perimeter of the cells after 3 h of culturing. Different letters above the columns in (b) refer to significant differences ($p < 0.05$) between different groups. H(−)L(−), treatment with pure water in a light-shielding box; H(+)L(+), 365 nm LED irradiation of sample in 3% H_2O_2.

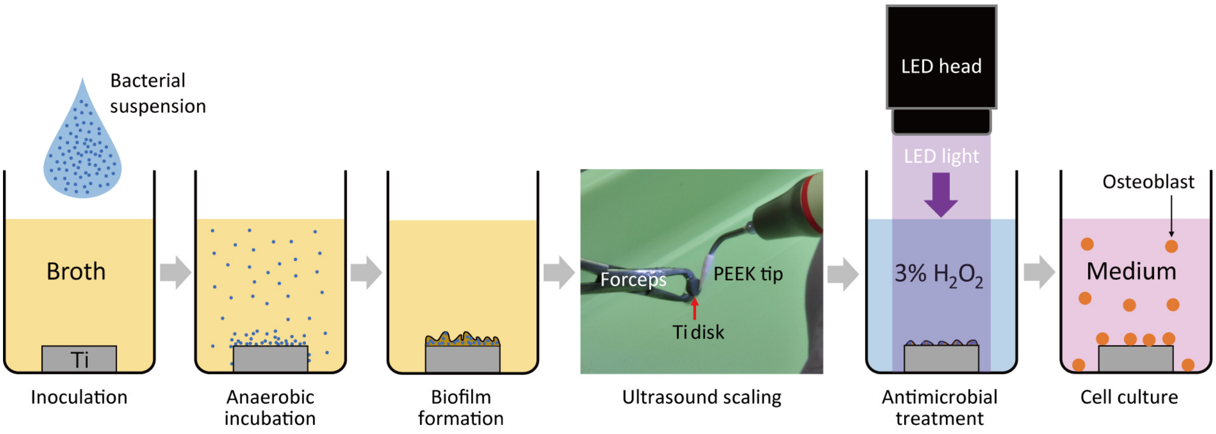

Figure S5. Schematic illustration of the study. A titanium disc was immersed in brain heart infusion broth or basal mucin medium inoculated with a bacterial suspension. After anaerobic incubation, biofilms formed on the titanium discs were mechanically eliminated via ultrasound scaling. Thereafter, the remaining biofilm was subjected to each antimicrobial treatment including H_2O_2 photolysis. Subsequently, MC3T3-E1 osteoblastic cells were seeded on the titanium disc.
